# Faecal Metaproteomic Analysis Reveals a Personalized and Stable Functional Microbiome and Limited Effects of a Probiotic Intervention in Adults

**DOI:** 10.1371/journal.pone.0153294

**Published:** 2016-04-12

**Authors:** Carolin A. Kolmeder, Jarkko Salojärvi, Jarmo Ritari, Mark de Been, Jeroen Raes, Gwen Falony, Sara Vieira-Silva, Riina A. Kekkonen, Garry L. Corthals, Airi Palva, Anne Salonen, Willem M. de Vos

**Affiliations:** 1 Department of Veterinary Biosciences, University of Helsinki, Helsinki, Finland; 2 Department of Medical Microbiology, University Medical Center Utrecht, Utrecht, The Netherlands; 3 KU Leuven, Department of Microbiology and Immunology, Rega Institute, Leuven, Belgium; 4 VIB, Center for the Biology of Disease, Leuven, Belgium; 5 Valio Oy, Helsinki, Finland; 6 Translational Proteomics, Turku Center for Biotechnology, University of Turku and Åbo Akademi University, Turku, Finland; 7 Department of Bacteriology and Immunology, Immunobiology Research Program, University of Helsinki, Helsinki, Finland; 8 Laboratory of Microbiology, Wageningen University, Wageningen, The Netherlands; University of Illinois, UNITED STATES

## Abstract

Recent metagenomic studies have demonstrated that the overall functional potential of the intestinal microbiome is rather conserved between healthy individuals. Here we assessed the biological processes undertaken in-vivo by microbes and the host in the intestinal tract by conducting a metaproteome analysis from a total of 48 faecal samples of 16 healthy adults participating in a placebo-controlled probiotic intervention trial. Half of the subjects received placebo and the other half consumed *Lactobacillus rhamnosus* GG for three weeks (10^10^ cfu per day). Faecal samples were collected just before and at the end of the consumption phase as well as after a three-week follow-up period, and were processed for microbial composition and metaproteome analysis. A common core of shared microbial protein functions could be identified in all subjects. Furthermore, we observed marked differences in expressed proteins between subjects that resulted in the definition of a stable and personalized microbiome both at the mass-spectrometry-based proteome level and the functional level based on the KEGG pathway analysis. No significant changes in the metaproteome were attributable to the probiotic intervention. A detailed taxonomic assignment of peptides and comparison to phylogenetic microarray data made it possible to evaluate the activity of the main phyla as well as key species, including *Faecalibacterium prausnitzii*. Several correlations were identified between human and bacterial proteins. Proteins of the human host accounted for approximately 14% of the identified metaproteome and displayed variations both between and within individuals. The individually different human intestinal proteomes point to personalized host-microbiota interactions. Our findings indicate that analysis of the intestinal metaproteome can complement gene-based analysis and contributes to a thorough understanding of the activities of the microbiome and the relevant pathways in health and disease.

## Introduction

During the last decade, many phylogenetic and metagenomic studies have revealed the vast genetic diversity and coding capacity of the intestinal microbiome in human adults [1,2]. We all harbour hundreds of microbial species, each carrying several thousands of genes and hence the intestinal microbiome enhances our own coding capacity by at least a hundred fold. Intestinal microbes are involved in many crucial functions, such as metabolizing food components that escape uptake by the host, contributing to the degradation of mucus, and producing vitamins and a variety of metabolites, such as short chain fatty acids and other important signalling molecules. Overall, the interactions taking place between the host and intestinal microbes, as well as between the different species of microbes, may exert a profound impact on the host’s health [3,4]. The type and amount of microbes contributing to these actions differ substantially between individuals which means that the phylogenetic composition can be considered as kind of a subjective fingerprint [5,6]. However, metagenomic studies have shown only limited differences between subjects. Strikingly, while our microbiota varies phylogenetically, metagenomic analyses have revealed that at the highest functional level, such as in KEGG pathway maps, the functional potential of the microbiome of healthy individuals remains very similar [7,8]. However, this does not imply that there is a similarity in the actual activity of the microbiota in vivo, because this is affected by a variety of factors, including diet, host genetics, and health status.

Two well-established functional approaches have been exploited to extend the genome-scale analysis to activity based profiling: metatranscriptome and metaproteome analyses. Recent faecal and ileal metatranscriptomic investigations have indicated that there are pronounced differences at the gene expression level in subjects with comparable metagenomes [9–11]. However, bacterial and archaeal transcripts have short half-lives, in the order of seconds to minutes and hence faecal RNA may not accurately capture the biological processes taking place in the intestinal tract. In contrast to mRNA, proteins are more stable and may provide an interpretation of activities along the whole digestive tract. In the absence of global enzymatic or physiological approaches, metaproteomics, the study of all proteins in an ecosystem, has been applied to elucidate the actual function of the intestinal microbiota [12]. This technique makes it possible to monitor the activities of the host and microbes at the same time, and thus to study their mutualistic interactions [13].

So far, intestinal metaproteomic studies have been carried out with 20 healthy individuals at a maximum of two time points [13–16]. Functional differences based on proteins have been studied in a dozen patients with strong perturbing factors such as Crohn’s disease or antibiotic treatment, but no functional baseline for healthy subjects has ever been established [16–19]. In general, functional differences between healthy individuals and their variation over time have only been studied to a limited extent.

Here, we expand these earlier studies with a cohort of 16 healthy subjects who took part in a placebo-controlled probiotic intervention trial and characterized the dominant functionalities by conducting a comprehensive faecal metaproteome analysis. In this longitudinal trial, these subjects were followed for six weeks and a total of 48 different faecal samples were taken; before, during and after the placebo or probiotic intervention, resulting in three analysis time points. These were used to identify the temporally stable proteins in each subject, i.e. the individual core, as well as the shared functions, i.e. the common core, to define a metaproteomic baseline in healthy subjects. Moreover, we addressed the question of whether the microbial composition can explain the microbial activities i.e. converging vs. diverging in terms of composition and function.

## Materials and Methods

### Ethical statement

**Fig 1:**
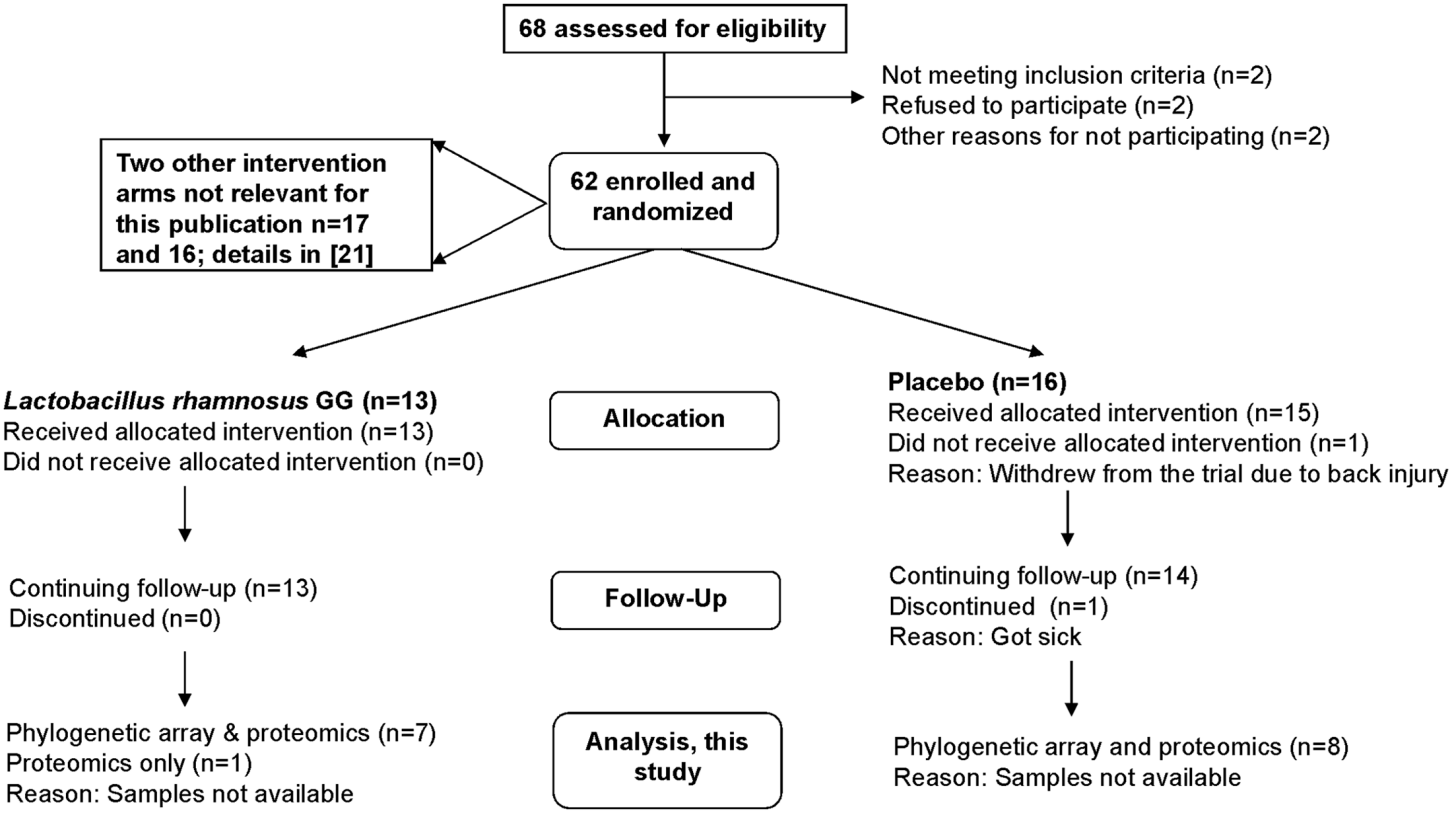
Consort flowchart of trial with ethical protocol no HUS 3577E0/05.

The Ethics Committee of the Hospital District of Helsinki and Uusimaa approved the trial and its protocol (Ethical protocol no HUS 3577E0/05; Fig 1). The trial is not registered in an international trial register. Written consent was given by the study participants prior to the start of the study. Details of this trial have been published previously [20–22].

### Study Design

The analysed samples were part of a randomized, double blinded intervention study testing the effect of various probiotic strains on immune and metabolic functions in healthy adults [21]. We focused here on the intervention with the well-described strain, *Lactobacillus rhamnosus* GG (ATCC 53103).

The trial consisted of a three-week run-in period, a three week intervention period and a three week wash-out period. Faecal samples at the end of each period were collected and stored at -20°C at home before transferring them within 24 hours to -80°C at the study centre. In this article, the sample collected at the end of the run-in period is called time point (TP) 1, the sample at the end of the intervention period is TP2, and the sample at the end of the wash-out period is designated TP3. During the intervention period study participants consumed each day 250 ml of a fruit based milk drink with or without *L*. *rhamnosus* GG (6.2 × 10^7^ cfu/mL amounting to a daily dose of 1.55 x 10^10^ cfu). In the present analyses we selected samples for which faecal material was left. Eight subjects of the placebo group and eight from the *L*. *rhamnosus* GG intervention group were included (S1 Table). The reduction of the study subjects due to sample availability resulted in a skewed age distribution between the placebo and LGG group (medians of 30 and 49 years, respectively). The median body mass index (BMI) was 23.9 in the placebo group and 25.9 in the LGG group. The female:male ratio was 6:2 in the placebo group and 5:3 in the *L*. *rhamnosus* GG group. As detailed in [21], trial participants kept a study diary. These included reporting any intestinal symptoms, the use of antibiotics and the regular use of probiotics (S2 Table). All individuals rated their health status as good, except for subjects 116 and 163 who described their health status as average.

### Protein extraction

Proteins were mechanically extracted from faecal samples (125 mg) by bead beating essentially as previously described [13]. Six cycles of bead beating (6.5 ms-1 for 45 seconds) were run on a FastPrep 24 (MP Biomedicals). The samples were kept on ice during the required 5 min resting period in-between the cycles.

### 1D gel electrophoresis and in-gel protein digestion

To reduce the complexity of the protein extract, the same 1D gel fractionation approach was carried out as described in [13]. The 37 kDa and the 75 kDa band of a prestained marker (Precision Plus^™^ Dual Color, Biorad) was used to define the height for cutting the lane into three fractions. Only the middle part (~37–75 kDa), containing the abundant metaproteome (AMP) was subsequently analysed. In order to control for possible run differences impairing reproducible cutting, one of the samples was run on all of the gels. Gel pieces were washed and proteins reduced, alkylated, and tryptically digested (per sample 200 ng of trypsin sequencing grade, Promega) overnight similarly as described in [23]. Following extraction from the gel-pieces, peptides were vacuum dried and stored at -20°C until used. Dried peptides were resolved in 2% formic acid and filtered through glass microfiber filters (Whatman; grade GF/C) for removal of gel particles. The peptide elutes were desalted by using OMIX tips (Agilent Technologies). After aspiring the samples 15 times, the peptides were eluted with 60% acetonitrile/2% formic acid, dried and reconstituted with 1% formic acid prior to LC-MS/MS.

### Liquid chromatography tandem mass spectrometry

Protein digests were analysed by LC-MS/MS on a nanoflow HPLC system (Easy-nLCII, Thermo Fisher Scientific) coupled to a LTQ Orbitrap Velos mass spectrometer (Thermo Fisher Scientific, Bremen, Germany) equipped with a nano-electrospray ionization source. The mobile phase consisted of water/acetonitrile (98:2 (v/v)) and 0.2% formic acid (solvent A) and acetonitrile/water (95:5 (v/v)) and 0.2% formic acid (solvent B). Ten μl of sample were injected into LC-MS/MS equipment. Peptides were loaded onto a reversed phase precolumn (in-house made, 2 cm long, 100 μm inner diameter) with solvent A and separated in a reversed phase analytical column (in-house made, 15 cm long, 75 μm inner diameter). Both columns were packed with Magic C18-AQ 5 μm 200 Å resin (Michrom BioResources Inc., Sacramento, CA, USA). Peptides were eluted by using a step-wise gradient (2–20% solvent B for 70 minutes and 20–40% solvent B for 30 minutes at a flow rate of 300 nl/min).

Full scan for eluting peptides was acquired in the mass range of 300–2,000 m/z of the Orbitrap-detector with 30,000 resolution at 400 m/z, AGC target set to 1000000 and maximum inject time set to 100 ms. Based on the results of the full scan, the 20 most intense ions were fragmented by CID fragmentation (normalized collision energy of 35%, activation time of 10 ms and activation Q set to 0.25) and scanned on the LTQ linear ion trap (AGC target set to 5000 and maximum inject time set to 50 ms). An isolation width of 2 m/z was used for precursor selection. Precursors whose charge state could not be determined or for which the charge state was +1 were discarded from the MS/MS analysis. Precursors were dynamically excluded for 30 s with a repeat count of one. Both MS and MS/MS scans consisted of one microscan. MS scans were acquired as profile data and MS/MS scans as centroid data. The peaks were extracted with the software proteome discoverer (Thermo Scientific) with default settings and the resulting Mascot generic format (mgf) files are available at ftp://MSV000079596@massive.ucsd.edu (generic password “a”).

### Peptide identification

Peptide identification was performed as described previously [13] with a database extended for the number of bacterial genomes and alternative human proteins (S3 Table). Peptide identifications were filtered with a false discovery rate (FDR) of 5%.

### Functional and phylogenetic annotation of peptides

Functional annotation of peptides was performed by a BLAST search of the original protein sequences against the Cluster of Orthologous Groups database (COGdb) as in [13]. In addition, a BLASTP 2.2.27 search of the original protein sequences against NCBInr was performed. In the KEGG Orthology (KO) retrieval, BLAST results were imported into the metagenomic data analysis software MEGAN [24] (version 5.1.5; default parameters: maxMatches = 100, minScore = 50.0, maxExpected = 0.01, topPercent = 10.0, minSupport = 50).

A possible phylogenetic origin was assigned to a peptide by the same principle, as described previously [13]. In brief, in order to achieve a successful taxonomic annotation a peptide was required to have a complete peptide match in the universal protein resource knowledgebase (UniProtKB) and in the case of multiple taxonomic hits per peptide the lowest common ancestor was reported. Instead of utilizing in-house Python scripts [13] the online tool unipept was used [25] (version 1.4; settings: I and L equated, advanced missed cleavage option (trypsinized peptides searched but results per miss-cleaved peptide combined), duplicates filtered).

In the quantitative analysis, spectra per peptide per class (COG, KEGG, phylogenetic level etc.) were summed and relative percentages per sample calculated for heatmap visualization.

To assess the contribution of distinct functionalities in a certain domain, genus, or species, the functionally and phylogenetically annotated spectra were queried for the taxonomic unit of interest. To create a subset of the data containing peptides with a human origin and to investigate the human functionalities in more detail, peptides with at least “Chordata” assignments were picked. Selecting only *Homo sapiens* specific peptides would have resulted in very few hits, due to the wide range of Chordata species with highly similar homologous sequences. Bacterial KOs were mapped onto microbial gut-specific metabolic modules (GMM), its syntax is provided in the supplement in [26]. The GMM presence is defined with a threshold of detection of more than two thirds of the KOs involved in one of the alternative metabolic pathways covered by a module (i.e. coverage > 2/3). The GMM abundance is defined as the median KO abundance in the maximum coverage alternative pathway, if coverage > 2/3.

### Rarefaction analysis

KO counts for the lowest functional level (i.e. fourth) were used for analysing how the numbers of found KOs change as a function of number of samples. All samples per individual were combined and the results of a rarefaction analysis including all possible combinations both for accumulated (KO rarefaction) and shared KOs (KO core) were plotted. The analysis was performed at intervals ranging from two to 16 samples. At each interval, 1000 bootstraps were taken from the samples, and the numbers of KOs present in one or more of the samples included in a bootstrap were counted. A similar analysis was performed by counting the number of KOs present in all of the samples included in a bootstrap. This resulted in an estimation of sampling distribution of KO counts at varying levels of sample numbers. The analysis was performed with R v.3.1.1.

### Phylogenetic analysis: microarray and quantitative polymerase chain reaction (qPCR)

Extraction of DNA was performed with the Promega kit; this involved homogenization of the faecal material with ample amount of 1x PBS and continuing DNA extraction with the pellet as described in [27]. A phylogenetic microarray (Agilent Technologies), the Human Intestinal Tract Chip (HITChip), was applied to analyse the phylogenetic composition based on 16S rRNA gene amplicons [28]. Hybridisation of the microarray and min-max signal normalization were performed as described [29]. Per target sequence the oligonucleotide probe signals were summed [30]. Throughout this study, relative abundances are provided at either the phylum- or the genus-like level. The genus-like level microarray data are provided in S4 Table. In addition to microarray analysis, qPCR of 16S rRNA regions was carried out to determine the total amount of bacteria, Bacteroidetes, *Bifidobacterium* spp. and *Lactobacillus* spp. present in 1g of faeces using taxa-specific primers as described previously [31]. The presence of archaeal DNA was analysed with qPCR, using primers specific for *Methanobrevibacter* [32].

### Statistical data analysis

The statistical programming language R was used for statistical data analysis [33]. Hierarchical clustering using complete linkage algorithm and the Jaccard distance between identified peptides was used to determine the dissimilarity of the metaproteome [34]. Hierarchical clustering (complete linkage) was also applied to the oligonucleotide profiles using 1-Pearson’s correlation coefficient (r) as the similarity measure.

Split violin plots (R package vioplot and http://mbjoseph.github.io/blog/2013/06/24/violin/) were used to visualize the distribution of relative abundances of bacterial phyla both for phylogenetic microarray and proteomic data.

To identify correlations between human and bacterial functional classes, Spearman correlation was applied, and the R package qvalue was used for calculating false discovery rate adjusted p-values (q-values). Results were filtered for a q-value < 0.05.

## Results

### Microbial and human proteins in the cohort reveal specific core functions

The analysis of the metaproteome of 48 faecal samples obtained at the three time points, from each of the 16 subjects, which had been selected from the probiotic and placebo groups (Fig 1), resulted in approximately 1 million mass spectra of which 213,855 (23.7%) could be identified (FDR < 5%) (S5 Table). Because of the ambiguity in mapping the peptides to distinct proteins—the so-called protein inference—that is especially noticeable in metaproteomics, we have compared samples based on their spectral counts per functional group and not per protein [12,35].

Functional annotations provided a COG or KO assignment for 78% of the identified spectra. In addition, we could assign a taxonomic origin according to the lowest common ancestor principle to 66% of the identified peptides, of which 80.9% were classified as bacterial, less than 1% as archaeal, and 13.8% as eukaryotic. The remaining 5.3% could not be assigned to a single domain as the peptides could be found in at least two domains of life. The differentiation into bacterial and eukaryotic/human peptides was utilized for determining domain-specific functionalities.

Overall, 967 different bacterial KOs were identified. Rarefaction analysis of the functional diversity across the 16 individuals (S1 Fig) showed that more than half of these bacterial KOs were already covered when combining the analysis of three individuals. A core of around 200 KOs was identified in any pair of two subjects and around 100 KOs were found in each of the individuals.

**Fig 2:**
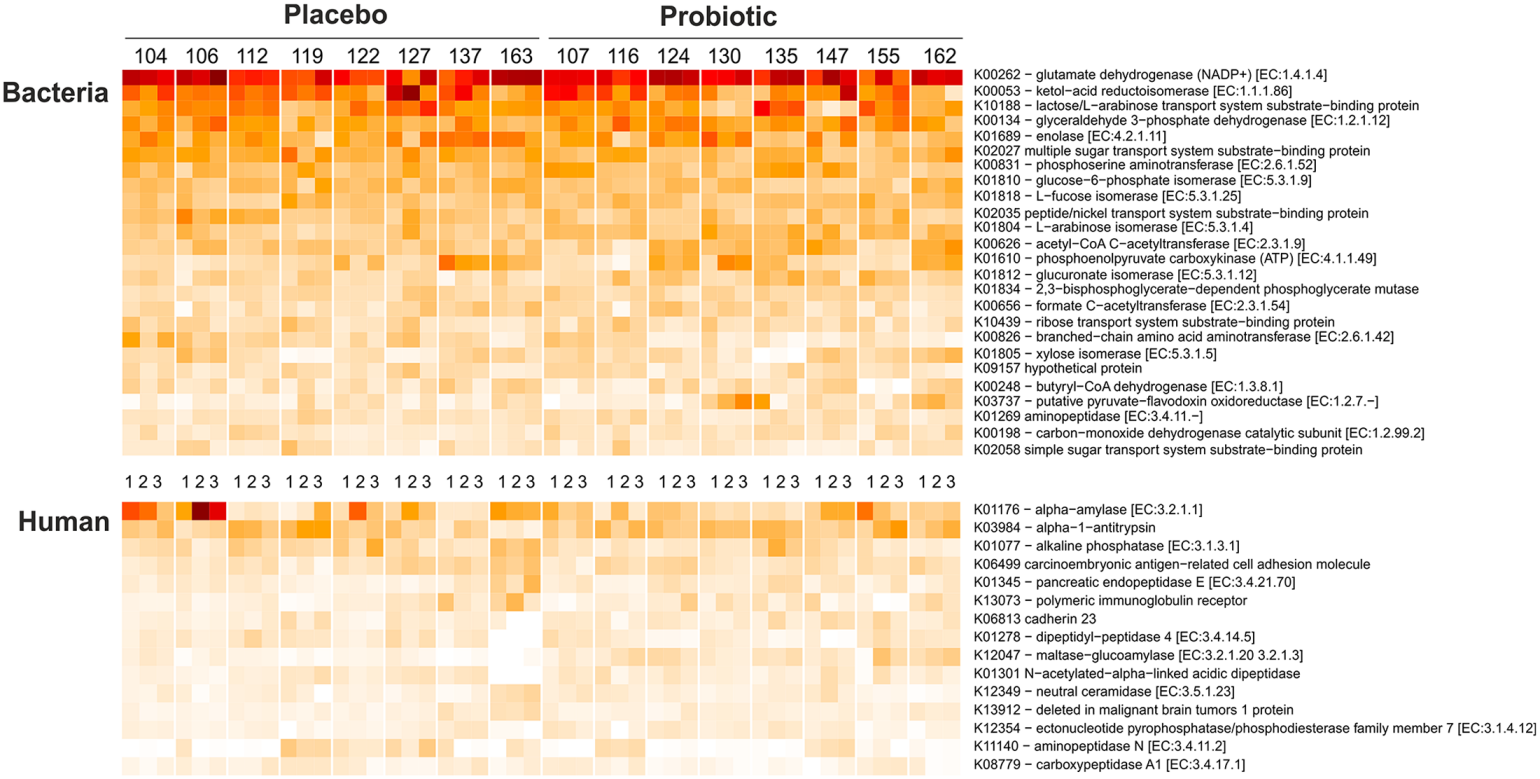
Top 25 bacterial (upper panel) and top 15 human (lower panel) functional categories (KEGG). (white to dark red range for bacteria KOs are from 0.1 to 10.2% out of 238 bacterial KOs and for human KOs from 0.0 to 49.3% out of 73 human KOs). The samples are organized according to treatment group (left: placebo group, right: probiotic group); sample time points are abbreviated as 1 (sample collected at the end of the run-in period), 2 (sample collected at the end of the intervention period) and 3 (sample collected at the end of the wash-out period).

**Fig 3:**
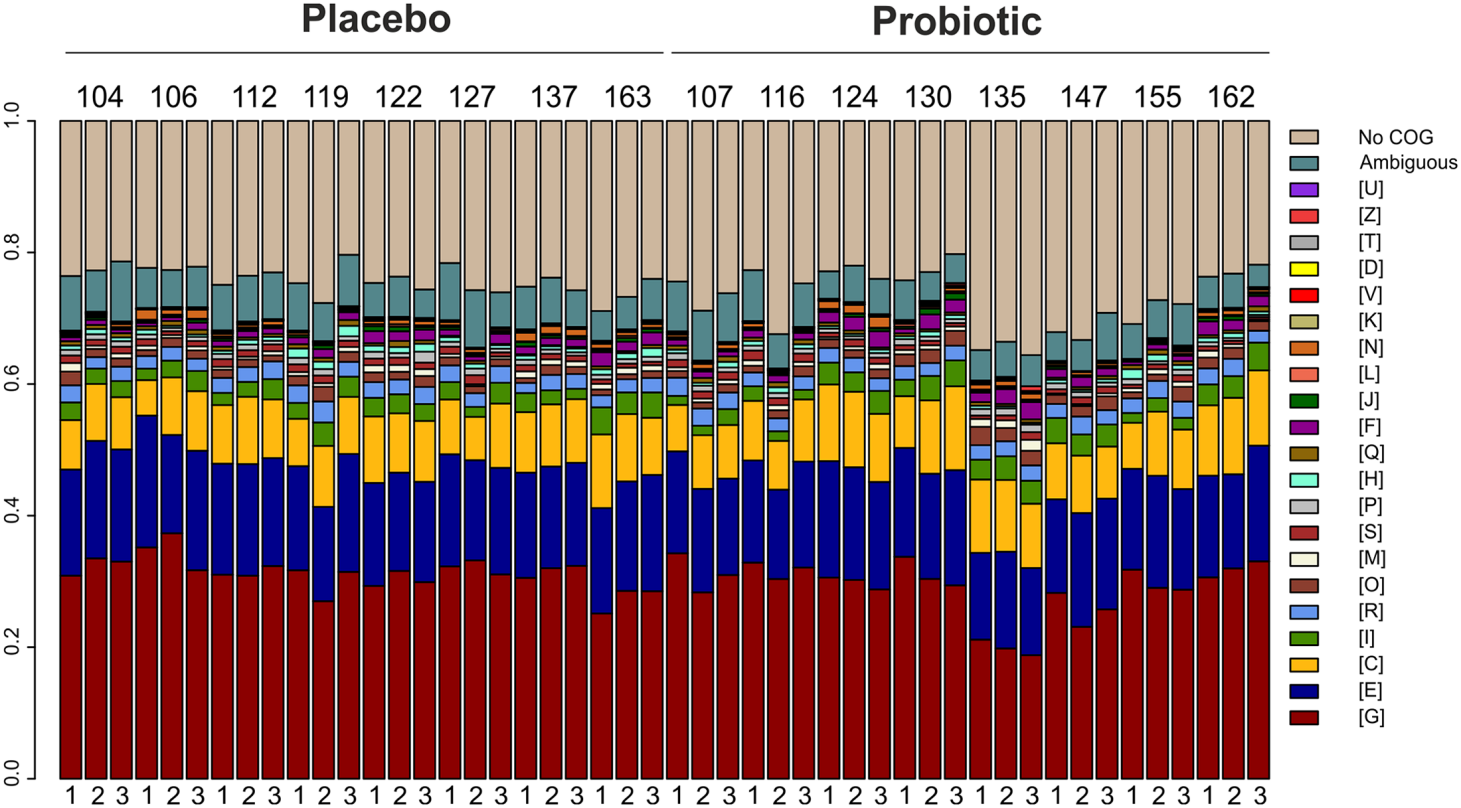
Relative distribution of COG families across samples. The samples are organized according to treatment group (left: placebo group, right: probiotic group); sample time points are abbreviated as 1 (sample collected at the end of the run-in period), 2 (sample collected at the end of the intervention period) and 3 (sample collected at the end of the wash-out period); ambiguous = functions being part of more than one COG family; [G] Carbohydrate transport and metabolism; [E] Amino acid transport and metabolism; [C] Energy production and conversion; [I] Lipid transport and metabolism; [R] General function prediction only; [O] Post-translational modification, protein turnover, and chaperones; [M] Cell wall/membrane/envelope biogenesis; [S] Function unknown; [P] Inorganic ion transport and metabolism; [H] Coenzyme transport and metabolism; [Q] Secondary metabolites biosynthesis, transport, and catabolism; [F] Nucleotide transport and metabolism; [J] Translation, ribosomal structure and biogenesis; [L] Replication, recombination and repair; [F] Nucleotide transport and metabolism; [K] Transcription; [V] Defense mechanisms; [D] Cell cycle control, cell division, chromosome partitioning; [T] Signal transduction mechanisms; [Z] Cytoskeleton; [U] Intracellular trafficking, secretion, and vesicular transport.

It was decided to concentrate on relevant intestinal functions and focus on those proteins present in the highest amounts. Therefore, we chose those KOs for which at least five spectra in one of the samples across the 48 samples had been identified (S6 Table). From these 238 remaining KOs, the 25 most abundant bacterial ones were identified in all of the samples, representing the most stable and abundant core functions (Fig 2). Glutamate dehydrogenase was found as the most abundant intestinal functionality (on average 7% of the spectra) in healthy individuals, as reported previously [13]. Other abundant proteins included major glycolytic enzymes as well as specific sugar-converting enzymes such as L-fucose isomerase, L-arabinose isomerase and glucuronate isomerase. The 238 bacterial KOs were mapped onto microbial gut-specific metabolic modules (GMM). A total of 21 out of 102 modules was detected as being enriched (S7 Table). With an average enrichment of 38 spectra the arabinose module was found to be extensively represented in all subjects at all time points. With respect to the COG family level, the metaproteomes appeared to be very similar (Fig 3). The COG families for carbohydrate transport and metabolism, energy production and conversion and amino acid transport and metabolism were the most representative families in all of the samples. Despite the rather even distribution of COG families across the samples, individual patterns were clearly discernible. These included a relatively higher level of COG family L (Replication, recombination and repair) in the samples of subjects 106, 137 and 124. Moreover, a clear difference could be observed in those peptides to which no COG family could be assigned, highlighting the importance of further studying these unassigned hits.

A total of 134 human KOs was identified. We selected those KOs with at least two spectra in one of the samples across the 48 samples, which resulted into 73 KOs (S8 Table). From these, the most abundant 15 KOs represented functions involved in digestion and host protection, and these showed specific patterns among all subjects and time points (Fig 2). Proteases and peptidases accounted for an average of 23% of the 73 most abundant human KOs. Carbohydrate and lipid-degrading enzymes were less abundant.

Correlation analysis of the human and bacterial KOs revealed 138 potentially relevant correlations (p-value and q-value < 0.05, S2 Fig). These included significant positive correlations between the human interacting serine/threonine-protein kinase 4 (K08848) and the bacterial flagellar hook protein FlgE (K02390), i.e. evidence for the presence of flagellins; between human alpha-amylase (K01176) and bacterial putative ABC transport system substrate-binding protein (K01989), propionate CoA-transferase (K01026), ATP-dependent Clp protease ATP-binding subunit ClpB (K03695), and cytidylate kinase (K00945), as well as a negative correlation between the antimicrobial protein DMBT1 (K13912) and the bacterial bleomycin hydrolase (K01372). Another indication for specific host-microbiota interactions may be deduced from the identification of an enrichment of degradation of the host-derived glycan mucin in at least two samples of four subjects (107, 119, 147, and 155) when mapping the bacterial KOs onto GMM.

### Evidence for a personalised and stable functional microbiome

**Fig 4:**
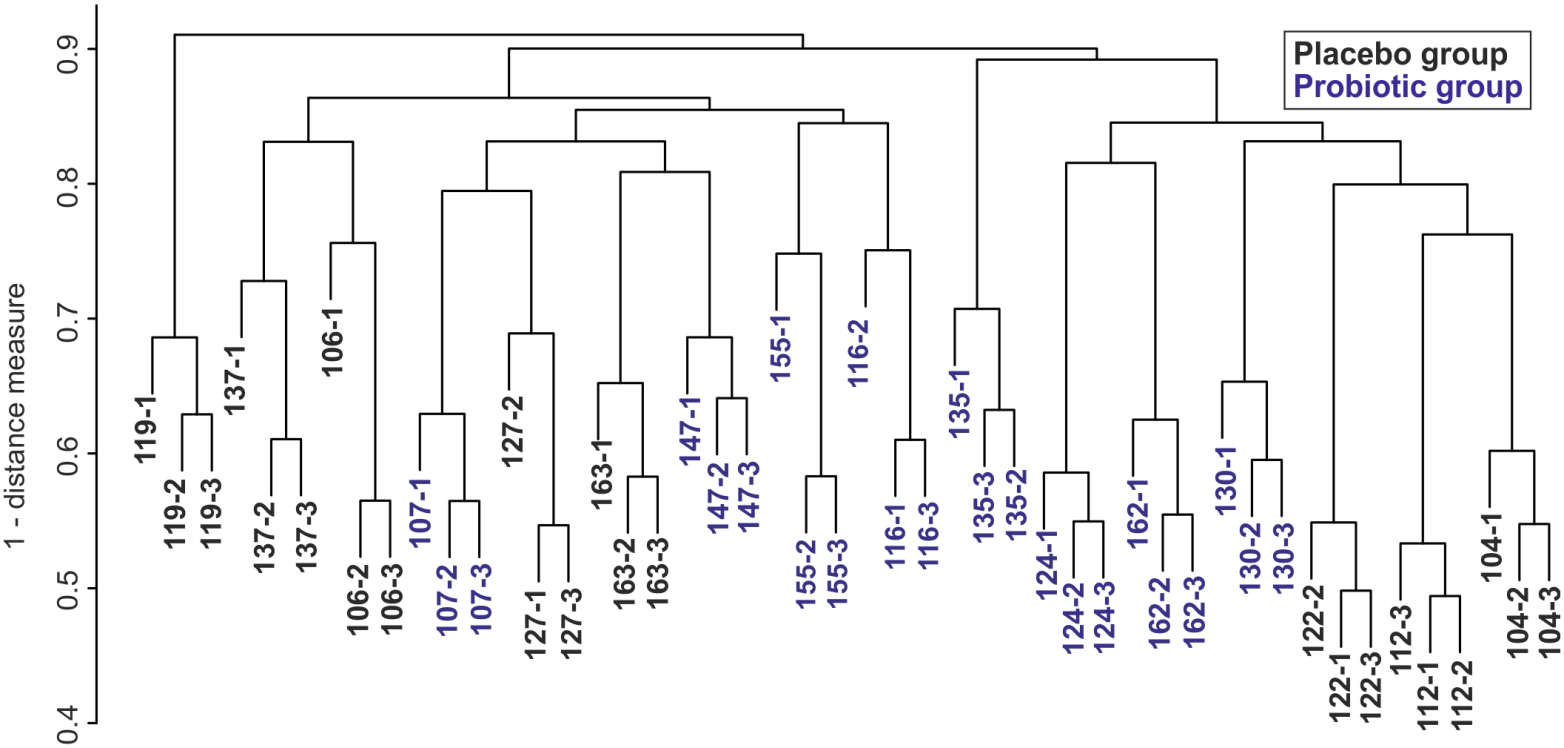
Hierarchical clustering of Jaccard similarity indexes of identified peptides. The peptide profiles clustered according to each subject however not according to the treatment group. The **s**amples from subject 119 differed the most and 135 the next most from the rest of the samples (at a distance measure of 0.9 and just below, respectively); labels: Subject—time point; 1: sample collected at the end of the run-in period, 2: sample collected at the end of the intervention period, and 3: sample collected at the end of the wash-out period; black font: placebo group, blue font: probiotic group.

A subject-specific clustering of all 16 subjects was observed when the identified peptides were used for hierarchical clustering based on Jaccard similarity (Fig 4). Moreover, also based on KO abundances (at least 5 spectra in one sample), virtually all of the samples clustered according to the individual subject (S3 Fig). This indicates that the functional microbiome is personalised and represents an extension of the compositional individuality that has been described previously and is now confirmed here (S4 Fig). Similarly, when comparing the peptide-based and the phylogenetic oligonucleotide-based hierarchical clustering in more detail, we observed that the samples obtained from subject 135 were quite distant from the other samples. However, in the peptide hierarchical clustering the samples of subject 119 were even more different than those of subject 135. This indicates that the proteins of subject 119 are rather unique and may reflect an aberrant health status and/or antibiotic use, as reported by this subject (S2 Table).

**Fig 5:**
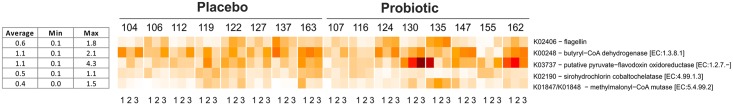
Heatmap of KEGG functions with marked differences between subjects. (white to dark red range for bacteria KOs are from 0.0–4.3% out of 238 bacterial KOs). The samples are organized according to treatment group (left: placebo group, right: probiotic group); sample time points are abbreviated as 1 (sample collected at the end of the run-in period), 2 (sample collected at the end of the intervention period) and 3 (sample collected at the end of the wash-out period).

To compare the individual metaproteomes on a functional level, we studied the individual differences of certain gut-specific KOs (Fig 5). The proteins involved in the production of short chain fatty acids (SCFA) are of interest due to the multitude of beneficial roles that SCFAs play in the intestine. The average levels for the KO for butyryl-CoA dehydrogenase, an enzyme involved in the production of butyrate, varied from 0.2% to 1.4% in the subjects. For methylmalonly CoA mutase, a key enzyme in propionate synthesis, levels varied from less than 0.1% to 1.2%. In general, the levels of KOs involved in butyrate synthesis were higher than those for proprionate (with butyryl-CoA dehydrogenase plus 3-hydroxybutyryl-CoA dehydrogenase and methylmalonyl-CoA mutase as proxies). For two subjects (119 and 155), similar amounts of both pathways were found (S5 Fig). Flagellins are signalling ligands of the toll like receptor (TLR) 5 and thus they represent microbial proteins that communicate with the host. This class of protein varied in the subjects from 0.2% to 1.6% (Fig 5).

The predominantly anoxic environment in the colon and other parts of the intestinal tract requires that the intestinal microbiota have to exploit anaerobic strategies for dealing with electrons. It was predicted that pyruvate-flavodoxin oxidoreductase might contribute to an anaerobic electron sink and this protein class showed a distribution varying from 0.4% to 2.9%. Sirohydrochlorin cobaltochelatase, an enzyme involved in the anaerobic biosynthesis of cobalamin (vitamin B12), contributed between 0.1% and 1.0% to the 238 most abundant KOs.

The consumption of LGG did not exert any effect on the identified peptides nor on the KEGG profiles (Fig 2; S3 Fig) i.e. the probiotic intervention did not lead to a systematic change of the identified peptides and their associated function. Since we tested this hypothesis with different statistical methods, we are confident that we did not detect functional differences. When calculating the ratio of bacterial to eukaryotic assignments, we did notice an increase during and a decrease after the intervention in the LGG group such that the samples from the LGG group clustered significantly together (S6 Fig; Pearson correlation; Fisher exact test p-value: 0.04). While this may point towards a decreased level of faecal host proteins in the LGG intervention group, stringent statistical analysis between the ratio of bacterial/eukaryotic peptides at time point one versus time point two in the LGG group did not reach statistical significance (p = 0.08).

### Variation in human proteins

The three most abundant human KOs were the starch-degrading enzyme alpha-amylase (average 11.7%), the serpin alpha 1 antitrypsin (10%) and the hydrolase alkaline phosphatase (6.1%); these revealed differences across and within individuals (Fig 2). The levels of alpha-amylase differed substantially between individuals. Relative abundances reached almost 50% at TP2 and TP3 in subject 106, but were consistently low (4–6%) in subjects 135 and 137. Notably, low alpha-amylase levels co-occurred with high levels of *Prevotella* spp. (see below).

High levels of alpha 1 antitrypsin of 10% were found in the majority of the samples with a minimum of 4.5% and a maximum of 18.4% of the human spectra.

### Phylum origin of 16S rRNA genes and peptides

A phylogenetic 16S rRNA gene based microarray (HITChip) was used to assess the microbial composition of the studied faecal samples. The average diversity (Shannon index) was 5.89 (min: 5.54, max: 6.3) in line with normal diversity observed in adults as observed in the in-house HITChip database with more than 1000 adult samples, which has been described previously [36]. The oligonucleotide profiles clustered according to subjects (S2 Fig). However, temporal variation was observed both at the phylum- and genus-like phylogenetic levels (S9 Table).

**Fig 6:**
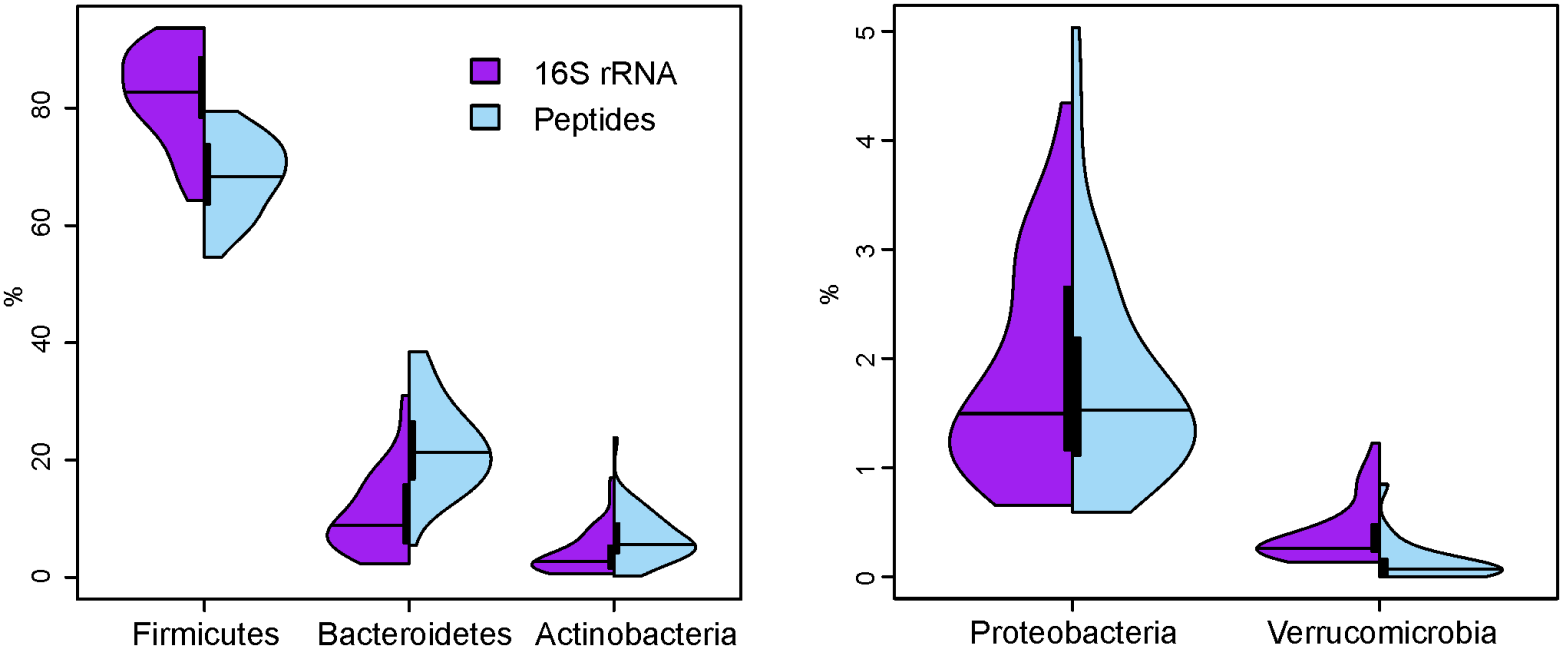
Distribution of the five main phyla in the studied cohort on 16S rRNA and peptide level. The split violin plots present side by side the relative amount (in percentage) of Firmicutes, Bacteroidetes, Actinobacteria, Proteobacteria and Verrucomicrobia obtained by 16S rRNA gene analysis (violet) and the metaproteomic measurements (blue). Both distributions are based on all 44 samples for which both 16S rRNA and metaproteomics data were available. The lines denote the median of the distributions (based on all the measured samples with the two different techniques) and the bar represents the 0.25 to 0.75 quantile.

On average, more than 99% of 16S rRNA gene signals and MS/MS spectra with microbial assignment could be assigned to the four phyla Firmicutes, Bacteroidetes, Actinobacteria, and Proteobacteria in descending abundance (Fig 6; data per sample in S7 Fig). The three main phyla varied extensively throughout all samples for both measurements. For example, Actinobacteria varied from below 1% to over 15% both for 16S rRNA gene and proteomic measurements. When compared to the 16S rRNA gene data, there were less Firmicutes MS/MS assignments but more Actinobacteria and Bacteroidetes MS/MS assigned. The distribution of Proteobacteria was rather similar with the HITChip and proteome data. Verrucomicrobia, the fifth most abundant phylum, had a slightly higher representation on 16S rRNA gene than at the peptide level. Looking at the ranking of the phylum the microarray and metaproteomic analyses agreed with the order of Firmicutes, Bacteroidetes, Actinobacteria, Proteobacteria, and Verrucomicrobia.

A high correlation (Pearson correlation coefficient of 0.67 at a q-value below 0.05) was found between phylogenetic microarray signals and spectral counts for Actinobacteria. Bacteroidetes and Firmicutes correlated negatively, at the phylogenetic microarray level as well as at the peptide level. However, in general, the Bacteroidetes peptide assignments were higher than the 16S rRNA gene measurements and only in a few cases was the phylogenetic array signal higher than the ratio of assigned peptides (S7 Fig). In addition, the Bacteroidetes microarray signals displayed more differences between the three time points, whereas the Bacteroidetes levels determined by taxonomic assignment of the identified peptides were rather stable. To control for possible bias originating from the microarray measurements, Bacteroidetes qPCR was carried out and these results confirmed the phylogenetic microarray data. The abundance of the most common intestinal methanogen i.e. the Euryarchaeon *Methanobrevibacter smithii*, was estimated by performing qPCRs; in 12 of the 44 samples more than 10^8^ 16S rRNA gene copies/g of faeces were detected. For five of these 12 samples, the Euryarchaea-specific peptide assignments were higher than 0.1% of the phyla assignments. In general, within 10,000 peptides with an assigned taxonomy, only about four Euryarchaea peptides were identified, with the exception of sample 135–3 in which almost 1% of peptides with a phylum assignment were of Euryarchaeal origin. Fungal peptides were identified at a similarly low level and around 4 out of 1000 spectra with taxonomic information were found to derive from plants (data not shown).

Plotting the COG assignments per phylum revealed a similar pattern as that previously observed with a smaller set of samples (S8 Fig) [13]. More than 59% of the Actinobacteria spectra were grouped to the category of Carbohydrate Metabolism, highlighting the impact of that phylum on sugar utilization and transport. Almost 50% of the Bacteroidetes spectra had no assigned function, emphasizing the further need for bacterial physiological studies to explore these unknown encoded functions.

### Pathways of prominent and distinct gut microbes

**Fig 7:**
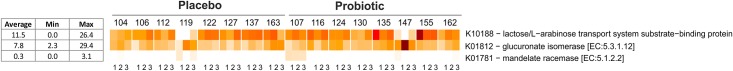
Heatmap of selected KOs assigned to *F*. *prausnitzii*. (white to dark red range for 0.0–29.4% out of 123 *F*. *prausnitzii* specific KOs). The samples are organized according to treatment group (left: placebo group, right: probiotic group); sample time points are abbreviated as 1 (sample collected at the end of the run-in period), 2 (sample collected at the end of the intervention period) and 3 (sample collected at the end of the wash-out period).

The most prominent microbial species in general (on average constituting 15% of the microbiota measured by 16S rRNA gene analysis) was *Ruminococcus obeum*, a well-known representative of the largest phylum Firmicutes. However, on average, only 1.6% of the bacterial peptide hits classified at least to a phylum assignment were specifically assigned to *R*. *obeum*, hampering comparisons between samples. In contrast, the second most abundant species based on phylogenetic data, *Faecalibacterium prausnitzii* (constituting around 10% of the microbiota assessed by microarray measurements), represented on average 9.8% of the spectra. Therefore, we further analysed the distribution of *F*. *prausnitzii* and its activities. Bacteria related to *F*. *prausnitzii* showed substantial variation across subjects and time points at the 16S rRNA gene level (S9 Fig). For some subjects, 16S rRNA gene and proteomics measurement displayed essentially the same trend (e.g. samples of subjects 104, 106, 112) but for some samples, the 16S rRNA gene and peptide measurements developed in the opposite directions. In particular, subject 137 was a standout example where *F*. *prausnitzii* was found to contribute less than 5% to the overall community, whereas its metaproteome exceeded more than 10% of all proteins, indicative of the very high gene expression activity of this bacterial group. The majority of the *F*. *prausnitzii* KOs were involved in amino acid metabolism, but almost half of the *F*. *prausnitzii* peptides could not be designated to any specific function. The analysis of *F*. *prausnitzii*’s functional variation showed a certain trend within individuals (Fig 7). For example, glucuronate-isomerase was detected in relatively high amounts across all samples with few exceptions. In subjects 119 and 147, very low levels of lactose/L-arabinose transport system substrate-binding protein were observed.

For the second largest phylum Bacteroidetes species-specific spectra were much less abundant than for the above-mentioned dominant Firmicutes. This was also found for the spectra of *Prevotella* spp., which in contrast to *Bacteroides*, are believed to be more prevalent in individuals consuming fiber-rich diets. For example, *Prevotella* methylmalonyl-CoA mutase was found in all of the samples of subject 162, indicating stable *Prevotella*-specific propionate synthesis in that individual (S10 Table). *Prevotella* microarray signals were around the mean of 1% but for subjects 130 and 135 they were up to 20%. For all the samples of these two individuals, several hundreds of *Prevotella*-specific peptides were identified especially for carbohydrate degrading enzymes (S10 Table). In addition, low levels of human alpha-amylase were found in the samples of these individuals. This may hint that high amounts of complex carbohydrates were part of these individuals’ diets and left for *Prevotella* to degrade.

Since Actinobacteria, the third largest phylum, have a distinctive lifestyle in the intestine, i.e. specialization in the utilization of complex carbohydrates, we attempted to clarify whether there would be any detectable intra-individual differences within this phylum. We found that Actinobacterial xylose isomerase was missing from the samples from individuals 119 and 135. There was variation in the amounts of Actinobacterial beta-fructofuranosidase between individuals, for example, low levels were detected in subjects 107 and 135 (S10 Table).

Some differences were found in the abundances of Proteobacteria KO, the fourth largest phylum in this study. Proteobacterial thioredoxin 1 was found in all of the samples but at a very low level. A subject-dependent pattern was found for Proteobacterial taurine-pyruvate aminotransferase (S10 Table). This enzyme mostly originated from *Bilophila*, a genus found to be present in a large proportion of the population and which produces sulphide from taurine [37]. Taurine has multiple roles in humans, such as the formation of bile salts, and *Bilophila* spp. may be involved in a balanced turn-over [38].

In the samples with relatively high levels of archaeal proteins (from subjects 135-2/3, 162–3) KOs involved in methane metabolism were detected, most prominently the methyl-coenzyme M reductase beta subunit (S11 Table).

## Discussion

Metaproteome analysis is a recently developed approach to identify at the protein level the highly expressed genes in an ecosystem [12,39].

Here, we studied the faecal metaproteome of 16 healthy Finnish adults who took part in a probiotic intervention and consumed a fruit-based drink with or without *L*. *rhamnosus GG* by sampling at three time points over a period of six weeks and found that the functional microbiome is both personalized and stable over time. Addressing bacterial, archaeal and human proteins, we were able to provide a state of the art overview of the activities of both the intestinal microbiota and the host.

### Functionality of the gut ecosystem

Around 10% of the identified microbial KO functions were found to be present in all the individuals; these represent the core of the intestinal ecosystem with carbohydrate transport and metabolism, energy production and conversion and amino acid transport and metabolism being the most prominent metabolic routes. Obviously, by analysing the metaproteome of 16 subjects there is not yet a saturation of identified functions but by adding more individuals more functions will still be identified. The identification of several sugar-converting enzymes in the core metaproteome is indicative of the microbiome’s ability to exploit glycans as a rapidly available energy supply. The observation that the arabinose module was found to be enriched in all individuals might be attributable to the fact that Finnish subjects are known to consume large amounts of fibres, specifically those derived from rye, which also contains arabinose [40–42].

Similar to the microbial core functions, also a core of the host functions could be detected. The most prominent biological activities found in the host core were proteinases and peptidases, indicative of high intestinal proteolytic activity or the high stability of these proteins, since only free amino acids and di- and tri-peptides are transmitted to the blood stream in the intestine. However, these proteinases and peptidases may also be acting on different routes and may possess signalling activities.

Other host core proteins included important functions such as the starch-degrading enzyme alpha-amylase (average 11.7%), the serpin alpha 1 antitrypsin (10%) and the hydrolase alkaline phosphatase (6.1%); these enzymes displayed differences not only between but also within the same individuals (Fig 2).

### Personalized and stable functional intestinal microbiome

We found that each adult contained a unique faecal metaproteome that was stable over the analysed period of six weeks. Therefore, compositional individuality was conserved at the functional level, suggesting that a distinctive set of microbes may be responsible for a specific distribution of functions. Further studies will be required to identify the factors involved. Most likely, host genes together with environmental factors (diet, drugs, hygiene setting) may prime the ecosystem to develop its individuality. The presence of a personalized functional microbiome was suspected based on peptide assignments and KO abundances. Thus, there seems to be a functional stable metaproteome and sequence differences (as detected at the peptide level) due to different microbial communities may not solely explain the clustering of the subjects. A comparison of the clustering at the oligonucleotide and peptide level could suggest that there is stronger individuality at the phylogenetic level in comparison to the metaproteomic data. However, this observation can be partly explained by the difference in the type of measurement and the subsequent difference in the applied distance measure (Jaccard similarity for peptide measurements versus Pearson’s correlation for microarray data). The developed metaproteomics approach is an a-priori non-defined approach i.e. identified peptides depend on sample (and not each time the very same peptides in one protein get analysed) whereas the microarray is an a-priori defined approach: i.e. each sample is tested against a fixed set of oligonucleotides.

Individual differences in functions can be very important for the intestinal ecosystem such as the observed large variations in butyrate production or flagellin generation (Fig 5). Notably, one subject (155) had low spectral counts for both butyryl-CoA dehydrogenase and flagellin, both assigned to the Firmicutes. Higher gene counts for flagellar assembly in combination with a higher abundance of members of the phylum Firmicutes were found in a Russian cohort when compared to a Western cohort [43]. The authors concluded that differences in diet i.e. a higher consumption of resistant starch in rural Russian areas, might account for these results. Glucuronate isomerase was among the most abundant 25 functional classes and showed variance between the individuals in this study; an earlier study described that the gene for this enzyme was present in similar amounts but differed highly in its expression levels across individuals [10].

We detected variations in the levels of an enzyme (sirohydrochlorin cobaltochelatase) involved in the anaerobic synthesis of vitamin B12. An accumulation of the genes involved in B12 cobalamin synthesis has been found in the intestinal microbiota of adults compared to infants [44]. Our data suggest that there might be factors other than age influencing vitamin B12 synthesis.

The observed differences in sugar metabolism, such as the absence of xylose isomerase derived from Actinobacteria in some subjects, may point to a diet lacking the respective substrates. However, as we do not have dietary records of the study participants, causal explanations for the individual as well as temporal differences between the faecal metaproteomes cannot be provided.

We did not detect any obvious functional differences in the faecal metaproteome following the probiotic intervention as compared to the placebo. This is in line with our previous results from the same cohort i.e. the LGG intervention did not change the overall composition of the faecal microbiota; only the amount of *Lactobacilli* (reflecting the ingested strain) differed significantly after the intervention [22]. With LGG representing only up to 0.1% of the total faecal community in the post-intervention samples, it is not surprising that LGG-specific proteins were hardly detected. However, immunological differences between the two groups have been detected [21], evidence that the probiotic product did exert some effects. Rather than colon that represents the predominant source of faecal material, it is more likely that differential protein expression after LGG intake takes place in the ileum which is a region of the gut less densely populated with bacteria and a more active site of signaling. It remains possible that the changes at the protein level in the gut lumen (versus intra-mucosal) are too subtle to be measured with the MS approach used here: i.e. data dependent MS analysis. However, there was a trend that the ratio of bacterial/eukaryotic peptides was increased in the LGG group.

Previously, comparative studies on faecal metaproteomes have been conducted between healthy and diseased subjects. These kind of studies in which an intact and pathologically perturbed intestinal ecosystems have been compared to each other represent much more drastic phenotypic differences than present in our study and may explain the lack of clear differences observed in our cohort [17,18]. In a comparison of the microbial profiles and metaproteomes of healthy and Crohn’s Disease (CD) patients, CD patients could be distinguished from the healthy controls based on their 2-DE proteome profiles [18].

### Human-bacterial interactions as assessed by metaproteomics

We were especially interested in intestinal proteins which could serve as a strong indication for the presence of host-microbiota crosstalk. Therefore, we correlated the spectral counts of human and bacteria derived KOs and identified several significant associations. These protein families included the pair of human derived receptor-interacting serine/threonine-protein kinase 4 and the bacterial flagellar hook protein FlgE. This could be one example of a human-microbe interaction in the gut as the human kinase is involved in signalling to elicit an inflammatory response for which flagellins, via TLR5 signalling, could be the stimulus. Human alpha-amylase correlated significantly with the functional families of bacterial putative ABC transport system substrate-binding protein, propionate CoA-transferase, ATP-dependent Clp protease and cytidylate kinase. These correlations could reflect the interdependence regarding nutrient resorption and utilization between host and microbes. Moreover, a negative correlation was observed between the antimicrobial protein DMBT1 (deleted in malignant brain tumors 1 protein) also termed SALSA and known to be present in large amounts in faeces already in early life [45], and a bacterial, amino-peptidase (bleomycin hydrolase). This finding may point to a physical human-bacterial-protein interaction or to an indirect interaction as agglutination of some other molecule to DMBT1 may suppress the expression of the aminopeptidase.

In addition to the identified correlations, we observed relatively high levels of L-fucose isomerase. As fucose is a typical component of many O- and N-linked glycans produced by mammalian species rather than by plants, its use by the intestinal microbiota is a further indication for a mutualistic interaction between host and microbes. Recently, modulation of the fucosylation of mucus proteins was claimed to act as a physiological mechanism to provide the microbiota with substrates in case of disease [46] and an absence of fucosyl transferase as is the case in non-secretor subjects, was associated with a distinctive intestinal microbiota composition with reduced levels of mucus-degrading bacteria, including *Bifidobacterium* and *Akkermansia* spp. [29]. Here, we could not identify any association between the abundance of L-fucose isomerase and any specific microbial groups.

### Activity of specific microbial groups

The rationale behind studying the metaproteome is that microbial composition and their gene content alone may not be sufficient to explain a certain phenotype. Therefore, we compared the metaproteomic data with the phylogenetic data generated from a microarray. In general, the observations based on the 16S rRNA gene and organism-origin of peptides were often in agreement. However, in some instances, this was not the case and this may reflect the level of biological activity of the specific microbial group. These detected differences between the microbial composition and protein-makeup add further evidence to earlier findings that phylogenetic profiles alone cannot reflect changes in the functions being expressed by the microbiome. In a study where metaproteomic profiles of healthy and CD patients were compared, differences in the amounts of certain proteins were observed although the abundance of corresponding species behaved in a different manner [18].

In this study, we found the amount of Firmicutes in our microarray data to be 1.2 times higher than that inferred from the metaproteomic data, even though the phylogenetic and proteomic measurements for the prominent representative species *F*. *prausnitzii* were in good agreement. These findings could indicate that Firmicutes proteins may be under-represented in the protein sequence database, so that they get relatively less identified, but *F*. *prausnitzii* is at least biologically as active as its presence would indicate. In our earlier study, the abundances for Firmicutes were the same but in contrast to the current findings we identified less Bacteroidetes at the peptide level than with the microarray [13]. The difference might be explained by the higher amount of samples. The ratios obtained for Bacteroidetes differ widely between studies partly due to different extraction efficiencies of the DNA extraction protocols being used [32]. The average relative abundance of 10% found here is at the lower end of the range reported for Bacteroidetes [47] but in the same range as in another Finnish cohort [5] and in a Chinese cohort [48]. We cannot exclude the possibility that the “overrepresentation” of Bacteroidetes peptides may reflect their potential underrepresentation in the DNA preparations as in our earlier study where we found relatively less Bacteroidetes peptides compared to the microarray data a different DNA extraction method, i.e. repeated bead beating, was applied. However, as the fluctuations seen at the microarray level were not observed at the peptide level, it might well be that we detected an actual difference in presence and protein production in Bacteroidetes. On the other hand, the observation may also be explained by the fact that extensive DNA transfer has been found in Bacteroidales species leading to a homogenous gene content across species [49] that may facilitate the identification of Bacteroidetes peptides in comparison to the more heterogeneous Firmicutes peptides. As the Bacteroidetes qPCR results corresponded well to the microarray measurements, a bias due to underrepresentation of certain taxa on the microarray seems unlikely.

The lower relative proportion of Verrucomicrobia in the MS/MS data versus 16S rRNA gene data may be due to an abundance issue, limiting the analysis of proteins of a phylum which contributed only around 1% to the overall microbiota. In an earlier study, we found a high number of Verrucomicrobia peptides but then also the measured DNA reads were well above 1% [15]. The peptide and qPCR results for Methanobrevibacter were also in good agreement with each other. The high correlation of the levels of 16S rRNA gene and protein measurement for Actinobacteria gave an indication for an association between the abundance and biological activity of corresponding taxa and also provided an assurance of the reliability of these measurements.

In summary, on one hand, our data allows us to conclude that proteomic data in general reflects phylogenetic composition but on the other hand, it highlights distinct activities and possibly to some extent, analytical bias such as cross-hybridisation and DNA extraction bias. Although today the taxonomic assignment of peptides is possible, due to the far from complete coverage in databases, the assignment is not perfect. Therefore, the technical and annotation issues noted here may explain the results to some extent; this is a limitation of all other meta-omics studies [50].

### Human proteins in faecal samples—relation to health

Specific human proteins in faeces have been used as markers for certain physiological conditions [51] but from a metaproteomic perspective, differences in gut-derived host proteins have not been studied extensively. Therefore, we also studied the variations in the proteins of the host as the applied approach identifies both microbial and eukaryotic peptides at the same time. We did not fractionate samples to yield a bacterial pellet, as that would selectively detect only those human proteins that are bound to bacterial cells [52]. Since changes of the host and microbiome proteome are being examined at the same time, in theory our approach makes it possible to investigate the mutualistic microbiome host relationship. Overall, the human KOs identified mostly involved in nutrient digestion and the immune response, a result in agreement with earlier metaproteomic studies [13,52]. On average, alpha-amylase, alkaline phosphatase and alpha 1 antitrypsin were the three top human proteins although there were extensive variations between subjects and time points. All of these three proteins have been linked to intestinal or extra-intestinal physiological conditions and they may also be one factor contributing to the individuality of the microbiota as discussed below.

The levels of alpha-amylase may have an effect on the substrate availability for certain microbial species living in the intestine. Recently, alpha-amylase was suggested to stimulate the growth of vaginal *Lactobacillus* spp. [53] by breaking down glycogen. In contrast, *Lactobacillus* strains from baby faeces have been found to exert inhibitory effects upon alpha-amylase and other carbohydrate hydrolases [54]. Therefore, differences in alpha-amylase levels may be reflections of different ecological niches favouring certain microbes. Alpha-amylase may also have a protective effect in the intestine in the same way as has been observed in the mouth where the enzyme inhibits the glucosyltransferase of *Streptococcus mutans* [55]. As observed in this study, the amounts of alpha-amylase have been found to vary with time but also to be consistently non-detectable in some individuals [13]. In a study of patients with diarrhea-type irritable bowel syndrome (IBS-D), elevated levels of tryptic activity and amylase were found [56]. Possibly there are higher levels of amylase during and after diarrhea as the rapid transit of chyme might lead to a decreased (bacterial) degradation of this enzyme. The finding that individual 104 had high levels of alpha-amylase after an episode of diarrhea is support for that hypothesis. In addition, other factors, such as diet, may have a role, since high alpha-amylase levels were also observed in other individuals who did not report any intestinal symptoms.

Tryptic activity in faecal samples has been associated with intestinal disorders such as IBD [57]. In general, proteinases have been intensively studied in the context of IBD and IBS [58]. The alpha 1 antitrypsin identified here may be involved in regulating the proteinase activities and maintaining a balance of protein degradation in the intestine.

In faeces of mice fed a high fat diet, the levels of several mouse proteins such as alpha 1 antitrypsin were higher in parallel with less identified microbial proteins in comparison to mice consuming a carbohydrate diet [59]. However, the same study also detected higher levels of chymotrypsin B and there was speculation that the proteinase inhibitors may act also through supplementary routes in addition to inhibiting proteinase activity. Therefore, the changes and differences in the human proteins observed here might be due to a certain diet on a specific day [60]. Alpha 1 antitrypsin has been associated with the treatment success of graft-vs.-host disease in GI transplantation in humans [61].

Alkaline phosphatase is a versatile enzyme [62]; in the gut it has been found to combat pathogen invasion into the intestinal epithelium [63]. In a mouse study, the presence of intestinal alkaline phosphatase promoted the growth of a variety of intestinal bacteria [64]. Variations between individuals in the levels of alkaline phosphatase have been detected previously [13]; here very high levels in one individual were found, and high levels of alkaline phosphatase were mostly identified in those subjects who reported intestinal symptoms. Elevated levels of alkaline phosphatase might be one way that compromised intestine attempts to restore equilibrium. It is tempting to speculate that the levels of this enzyme could be a marker of the “quality” (intact, perturbed or somewhere in-between) of the host-microbe interaction.

DMBT1 (deleted in malignant brain tumor) was also one of the top 15 human KOs, its levels were quite abundant in most individuals. As this is a healthy cohort, this result is not unexpected since it has been hypothesized that this protein, which is involved in host defence mechanisms, is indicative of a healthy intestine [52]. Many of the human KOs identified here including alpha 1 antitrypsin, carboxypeptidase A1 and B, and alkaline phosphatase have been found previously to be equally abundant throughout the intestinal tract when proteins at the luminal interface were analysed [65], reflecting their crucial roles in intestinal activity.

Our results suggest that although over time variations in human KOs may be due to pathology, diet and other environmental factors may also contribute to these variations. Inter-individual differences may be influenced by different genetic backgrounds, both human and microbial. It is evident that both human and bacterial proteins need to be studied to fully reveal the mechanisms in the complex ecosystem of the intestine.

## Conclusions

By analysing the proteins from faecal samples of 16 healthy individuals at three measurement time points, the obtained metaproteome was found to contain a functional core but also to show variation across individuals and with time. This study can be viewed as a first step towards defining a healthy baseline, which is essential before one can evaluate the metaproteomes of diseased individuals, similar to the proposal concerning metagenomics [66].

A larger, well-stratified study cohort will be required to address the question of which microbial composition leads to which metaproteomic phenotype, but based on our current data, we conclude that different microbial profiles can lead to differences in the functionality of the intestinal microbiota.

Assigning taxonomies to peptides revealed overall good correlations with the phylogenetic data but caution is required, as presently it is difficult to estimate whether under- and over-representation of proteomic data is due to the actual biological activity of the microbes or to experimental artefacts such as a (data) analysis bias (technical and annotation bias). Very few studies have compared the composition of the microbiota and its actual functions being carried out, but there is growing evidence that accessing the composition alone is not enough. It appears that while the phylogenetic differences across samples are quite pronounced, metagenomes remain more stable. However, when evaluating the functions being expressed, then the difference may again be larger.

Overall, the inter- and intra-individual differences appeared rather small at the level of COG families. This indicates that with a stable proportion of main functionalities, there is a “healthy balance”, e.g. in protein turnover and replication, in the intestinal ecosystem. Individual differences are more likely to be detected as one moves further down in the functional hierarchy.

This study is one step in the gradual exploration of the biological activity of our gut symbionts. However, before we have a fundamental understanding of the crucial mechanisms present in the intestine, it will be essential to clarify the functions of the many so far unidentified proteins. In addition, functional classification systems have to be optimized for the human microbiome. Overall, metaproteomics is still in its infancy and improvements, especially regarding analytical depth and data analysis, are needed (e.g. in assigning peptides to protein groups for quantitative comparisons) to fill in the large knowledge gaps which are an inherent limitation of functional meta-omic analyses.

The combination of phylogenetic and metaproteomic data helps to gain a better understanding of the intestinal microbiota. For example, it reveals which microbes are living in the intestine and how they contribute to the host’s metabolism including signalling to other bacteria and signalling to the host. In the future, longitudinal studies will be required to clarify the normal flux both in composition and biological activity of our microbes.

## Supporting Information

S1 Fig Rarefaction and core analysis of identified bacterial KOs. (TIF)

S2 Fig Correlation of human and bacterial KOs (Spearman). (PDF)

S3 Fig Hierarchical clustering of KO abundances. (PDF)

S1 File This file contains S4 Fig Hierarchical clustering of oligoprofiles, S5 Fig Distribution of butyrate and propionate synthesis, S6 Fig Clustering of Pearson correlations of bacterial/eukaryotic assignment ratios, S7 Fig A) Distribution of the 5 most abundant phyla for 16S rRNA gene and spectra per sample. B) Bacteroidetes qPCR results, S8 Fig Functionality (COG) across phyla, and S9 Fig Abundance and activity of *F*. *prausnitzii*.
(PDF)

S1 Table Subject Information. (XLSX)

S2 Table Health questionnaire. Supplementary results on metaproteomics findings in combination with health questionnaire information. (PDF)

S3 Table Composition of the in-house human intestinal metaproteome database. (DOCX)

S4 Table Microarray data on genus-like level. (XLSX)

S5 Table Overview of mass spectrometric results and taxonomic assignment. (XLSX)

S6 Table Identified bacterial KOs. (XLSX)

S7 Table Microbial gut-specific metabolic modules (GMM). (CSV)

S8 Table Identified human KOs. (XLSX)

S9 Table Genus-like taxa distribution. (TXT)

S10 Table Selected KOs of Proteobacteria, Prevotella, Actinobacteria. (XLSX)

S11 Table Identified KOs from Archaea. (XLSX)
