# Supplementary figures and images for: Faecal Metaproteomic Analysis Reveals a Personalized and Stable Functional Microbiome and Limited Effects of a Probiotic Intervention in Adults
Source: PLoS One. 2016 Apr 12;11(4):e0153294. doi: 10.1371/journal.pone.0153294 (PMC4829149; doi:10.1371/journal.pone.0153294)

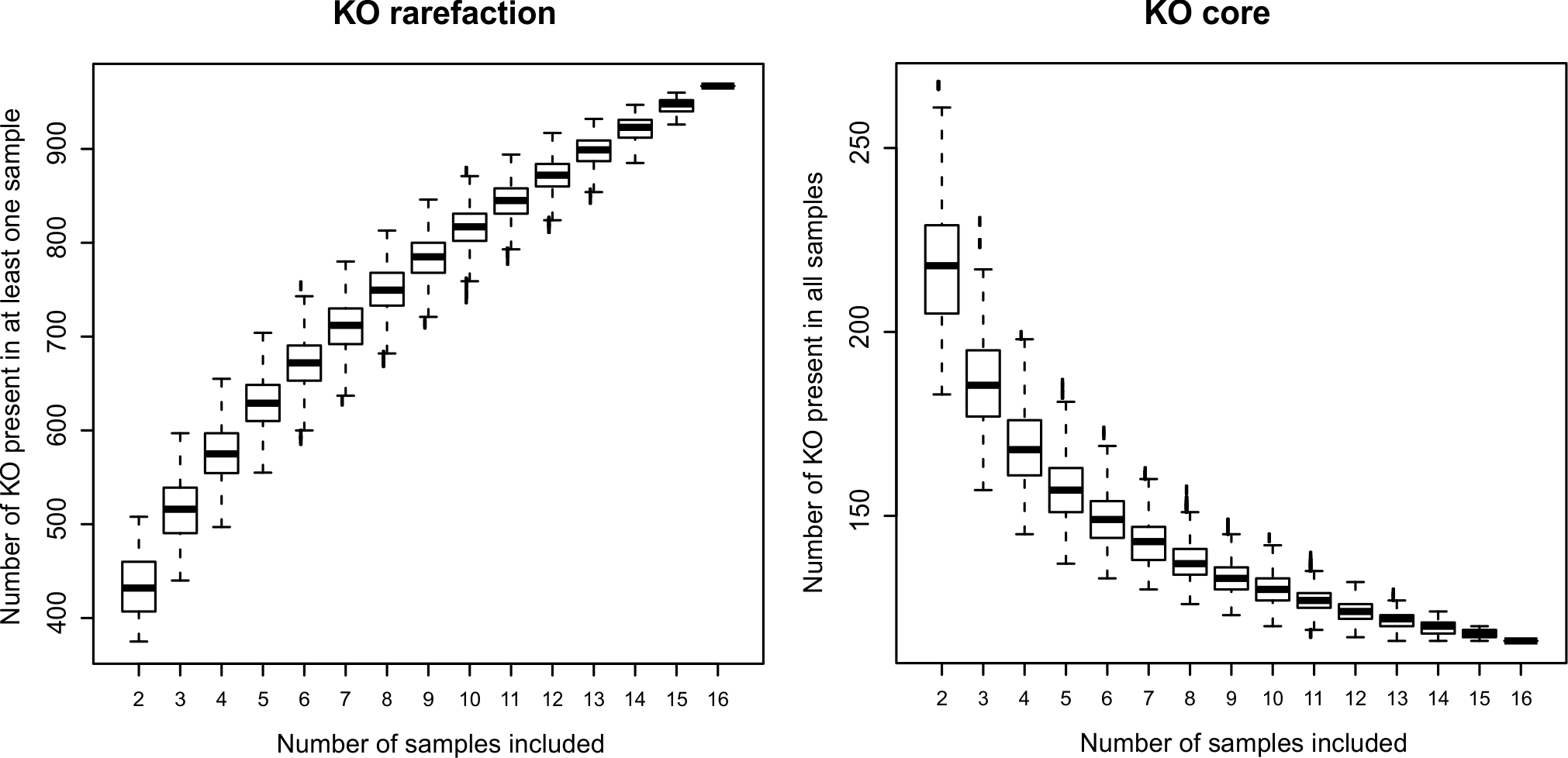

Supplement: S1 Fig — (TIF) [file pone.0153294.s001.tif]

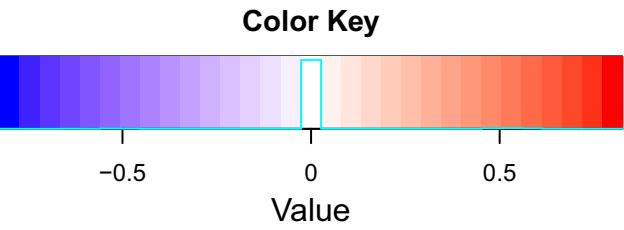

q-val< 0.05 abs(Cor)> 0

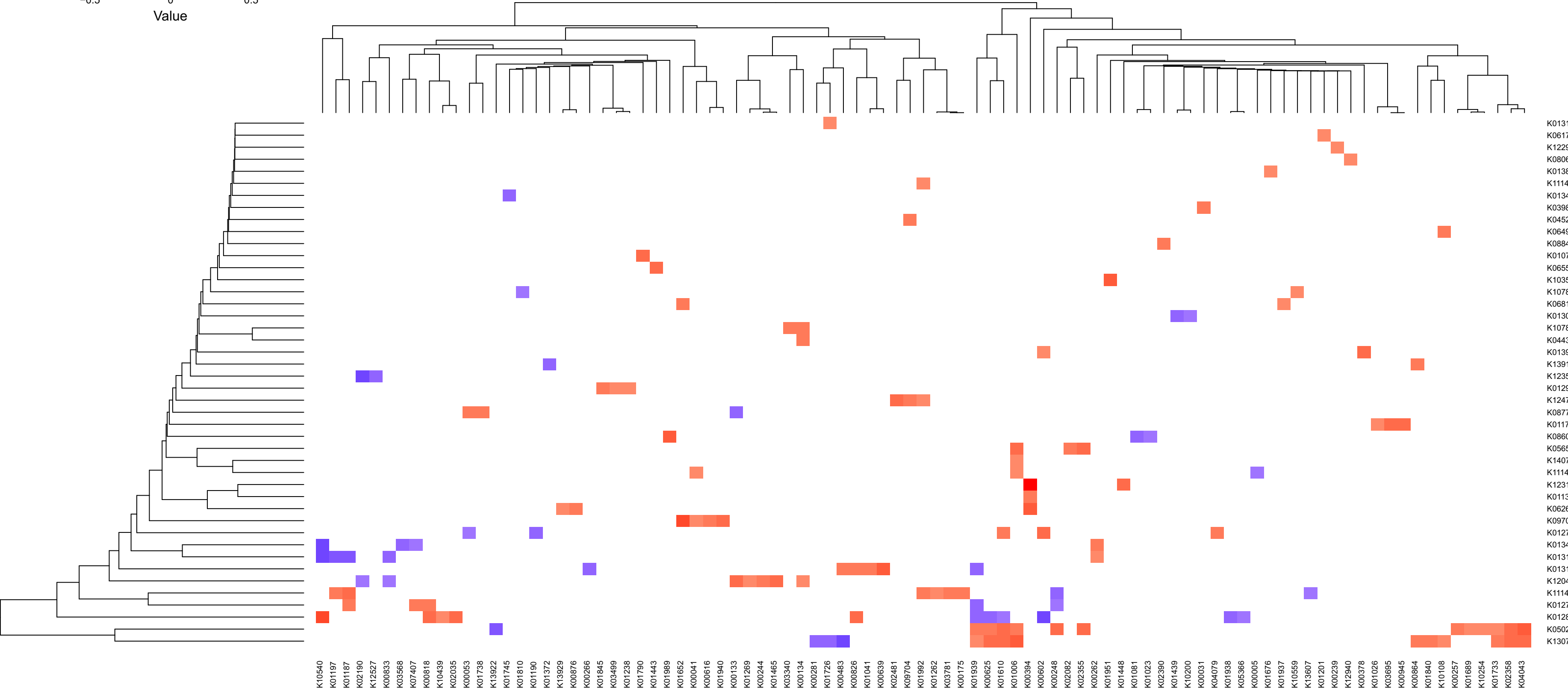

Supplement: S2 Fig — (PDF) [file pone.0153294.s002.pdf]

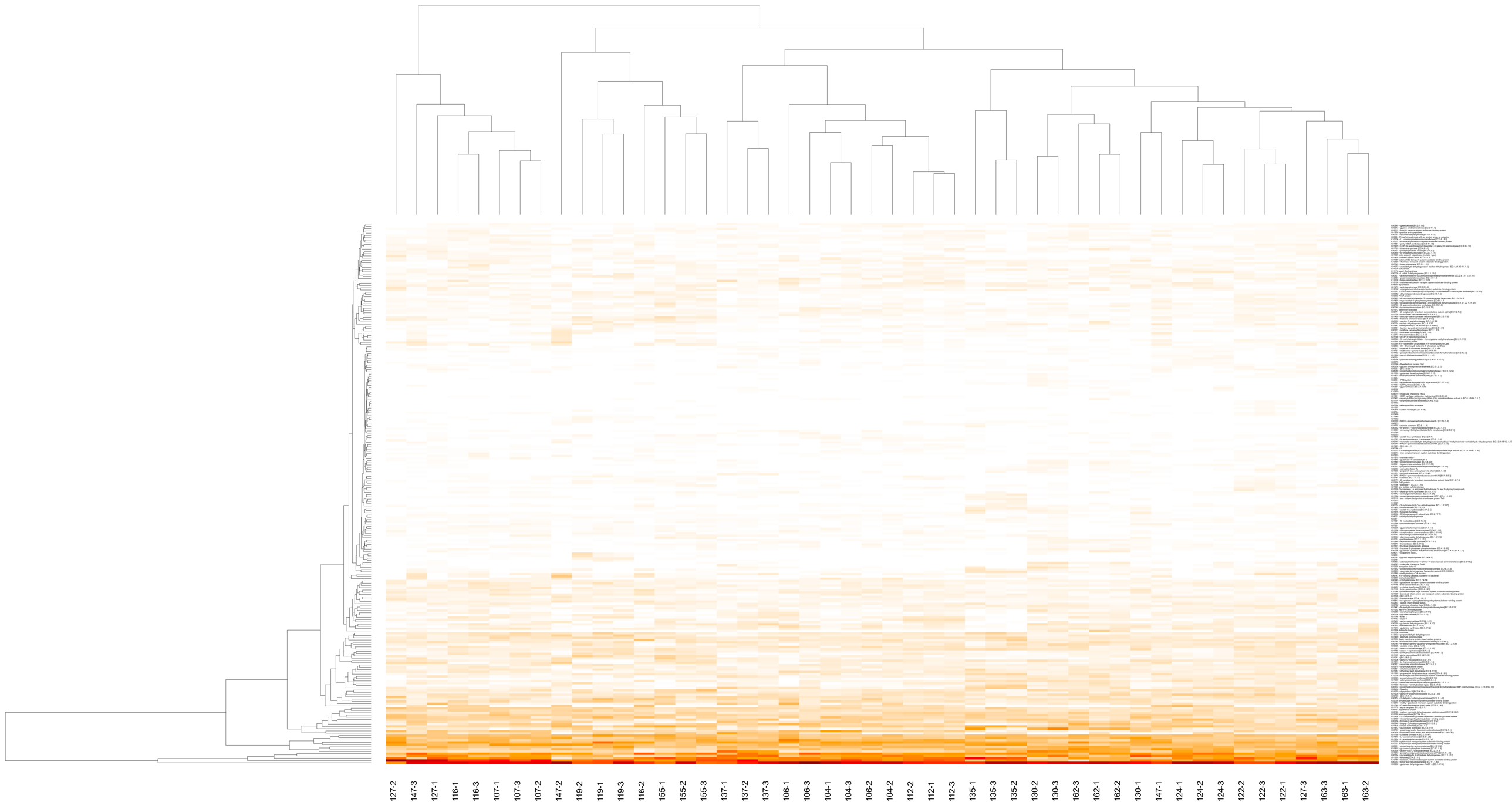

Supplement: S3 Fig — (PDF) [file pone.0153294.s003.pdf]
