# Supplementary material for: Faecal Metaproteomic Analysis Reveals a Personalized and Stable Functional Microbiome and Limited Effects of a Probiotic Intervention in Adults
Source: PLoS One. 2016 Apr 12;11(4):e0153294. doi: 10.1371/journal.pone.0153294 (PMC4829149; doi:10.1371/journal.pone.0153294)

S4 Fig. Hierarchical clustering of microarray data (oligoprofiles).

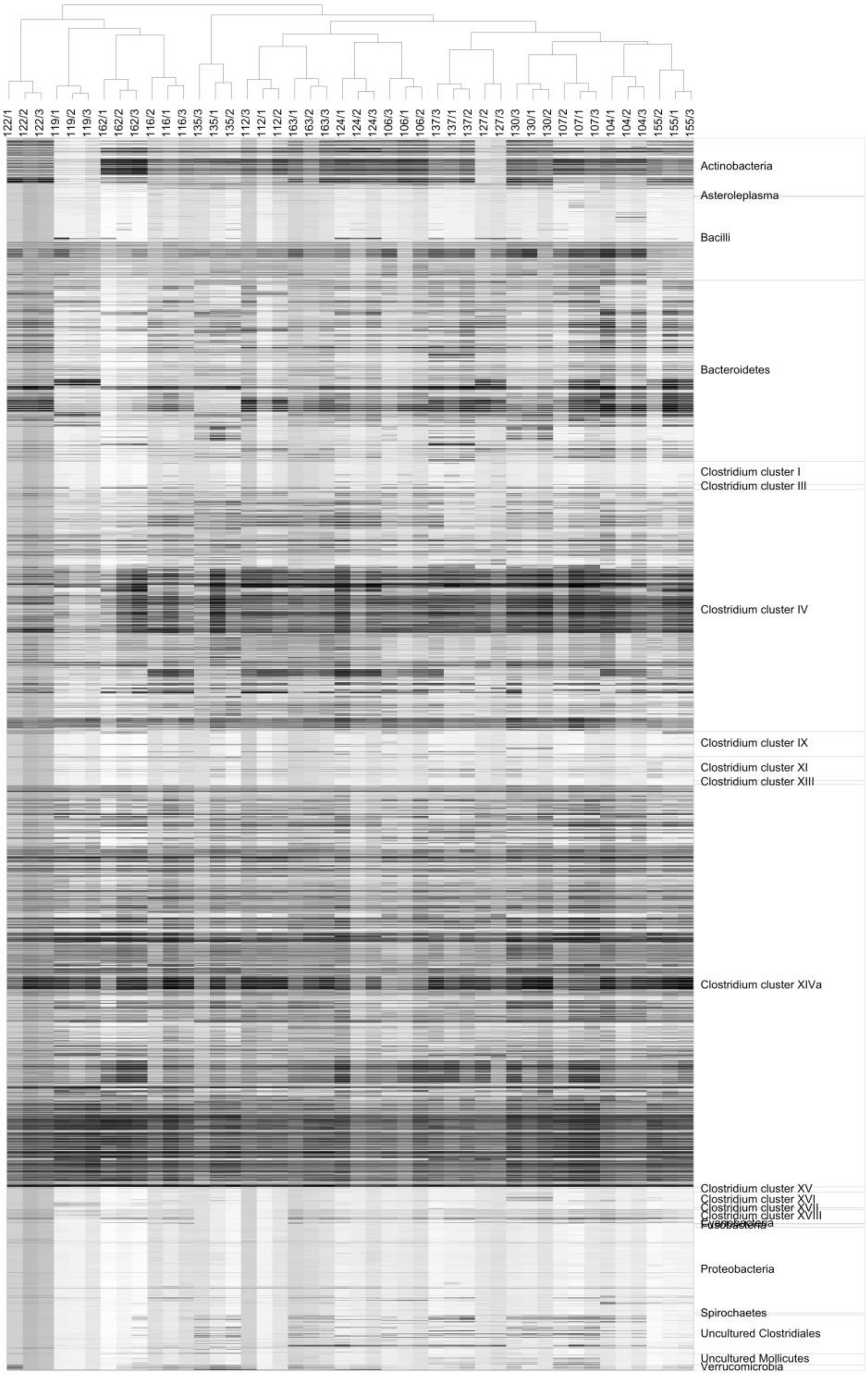

S5 Fig. Distribution (percentage out of 273 bacterial KOs) of butyrate (== butyryl-CoA dehydrogenase+3-hydroxybutyryl-CoA dehydrogenase) and propionate (methylmalonyl-CoA mutase) pathway. The samples are organized according to treatment group (left, 104 to 163: placebo group; right, 107 to 162: probiotic group).

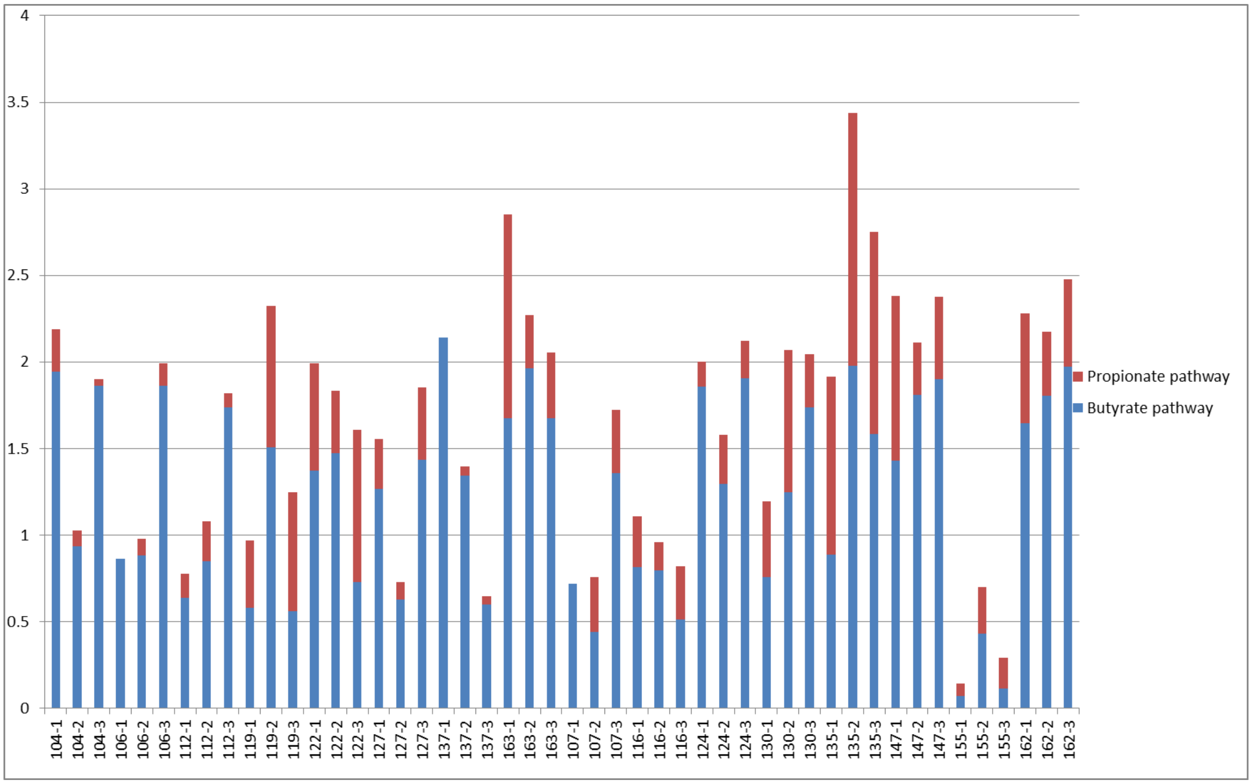

S6 Fig. Clustering of Pearson correlations of bacterial/eukaryotic assignment ratios. Performing a t-test for the difference in the bacteria/eukaryotic ratio between time-point one and two resulted in a p-value of 0.08 for the LGG group and 0.57 for the placebo group. Testing the difference between time point two and three resulted in a p-value of 0.17 for the LGG group and 0.10 for the placebo group. (time point one to three: 0.56 p-value LGG; 0.44 p-value placebo).

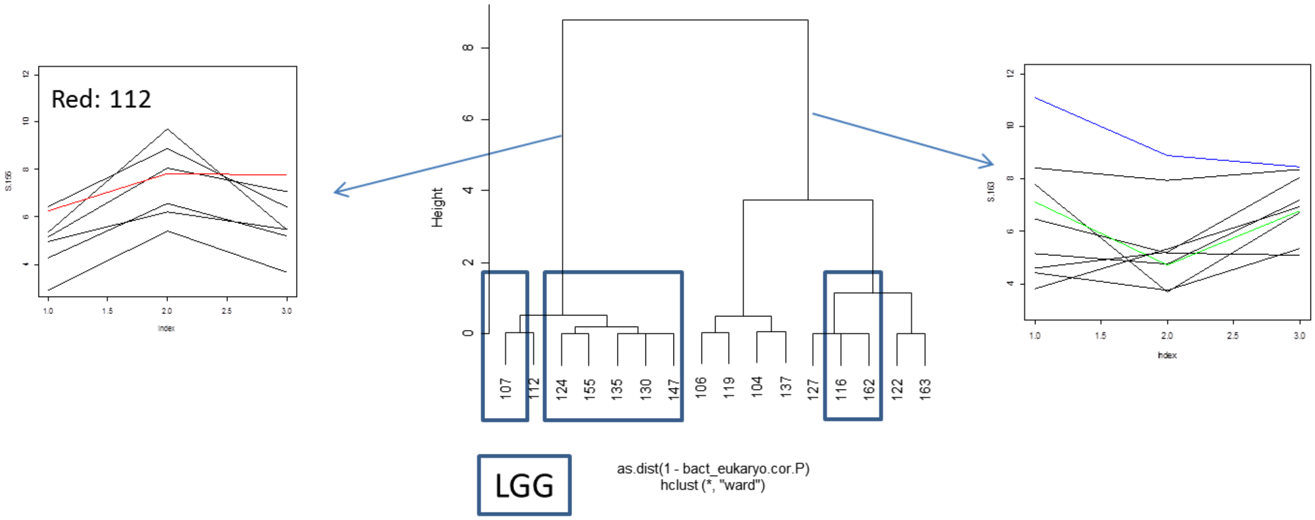

S7 Fig. A) Distribution of 5 most abundant phyla for 16S rRNA and spectra per sample. B) Bacteroidetes qPCR results. For 127-1 and all samples of 147 there was no HITCHip data available. The samples are organized according to treatment group (left, 104 to 163: placebo group; right, 107 to 162: probiotic group).

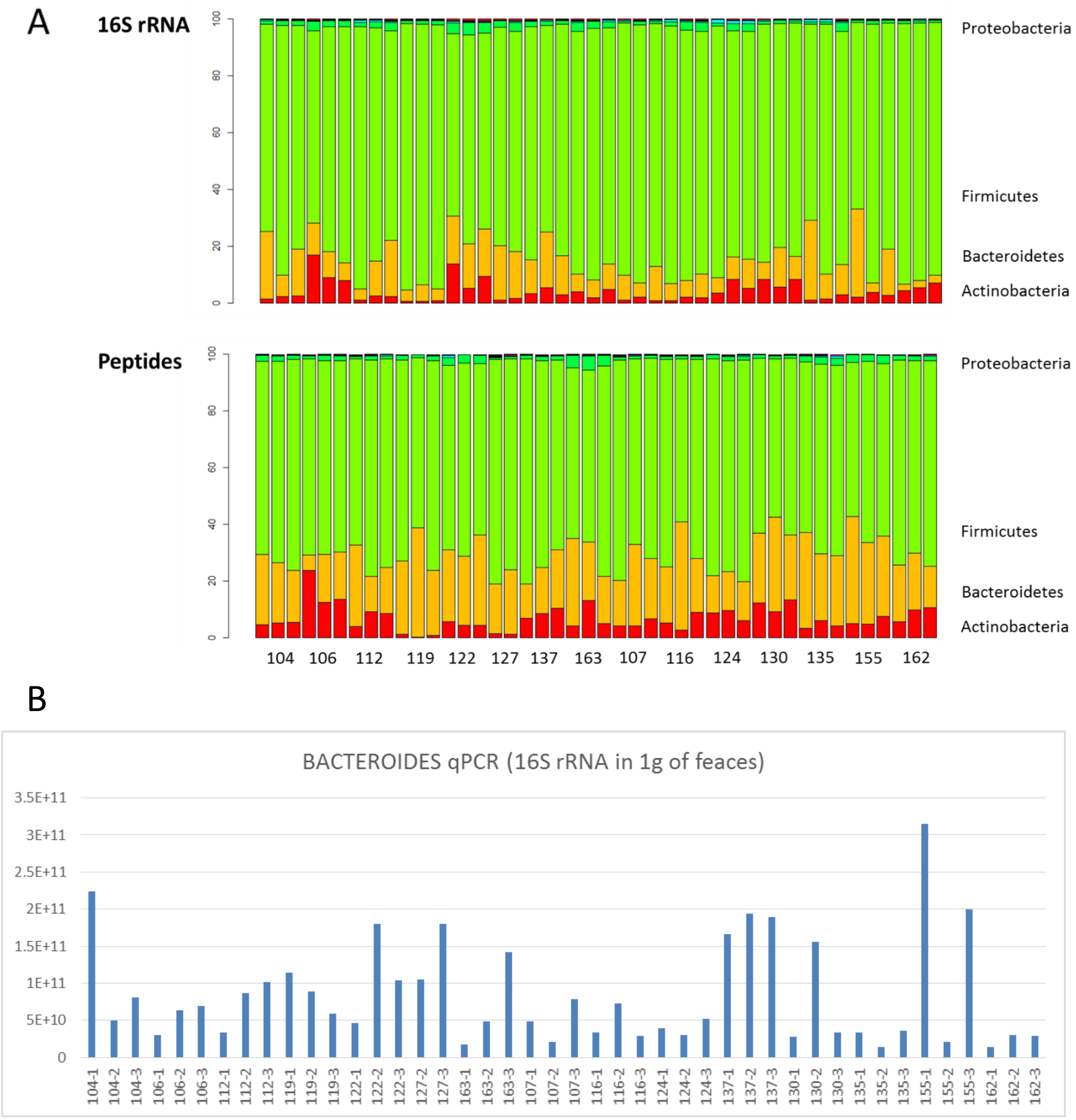

S8 Fig. Functionality (COG) across phyla. Ambiguous=functions being part of more than one COG family.

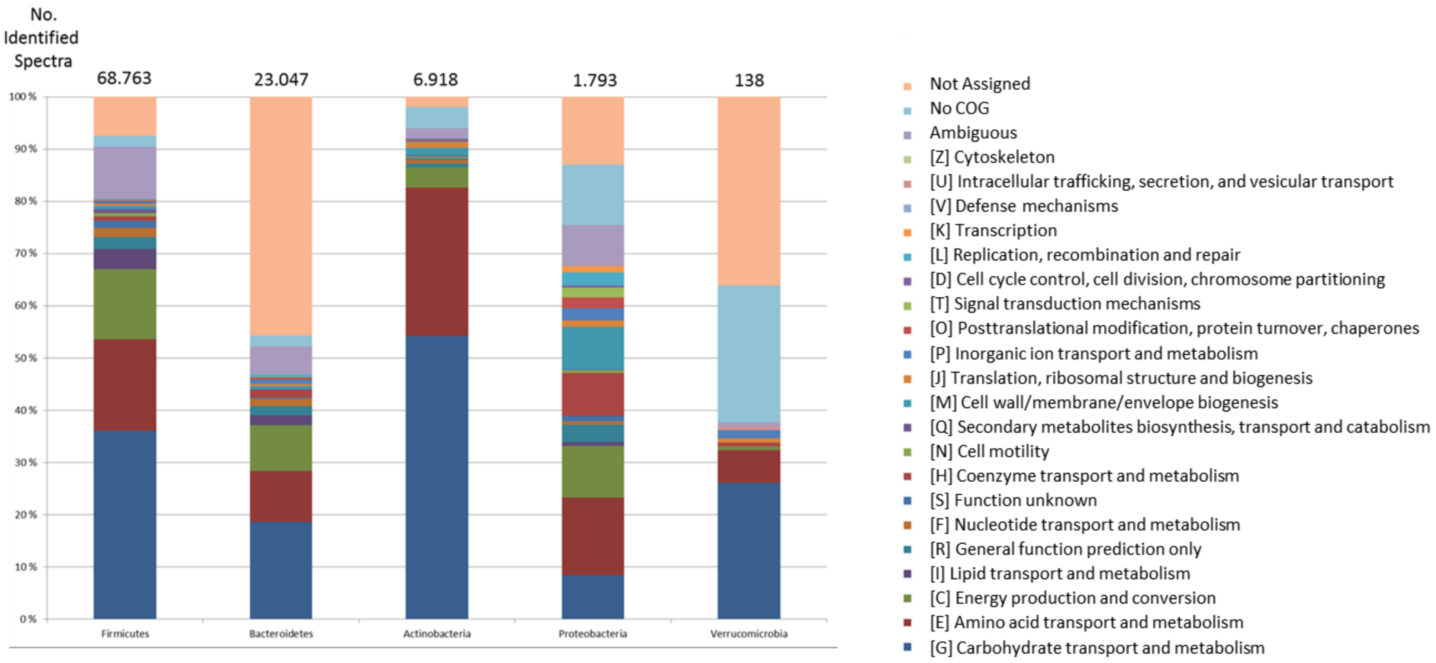

S9 Fig. Abundance and activity of *F. prausnitzii*. For 127-1 and all samples of 147 there was no HITCHip data available. The samples are organized according to treatment group (left, 104 to 163: placebo group, right, 107 to 162: probiotic group).

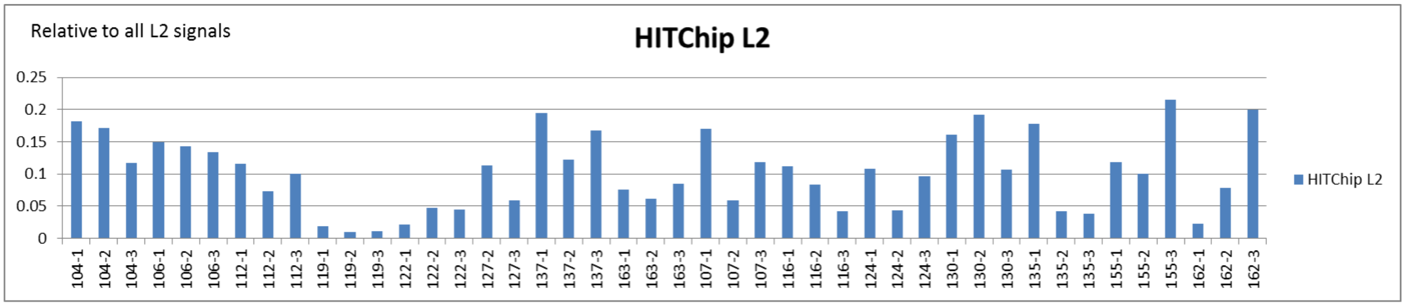

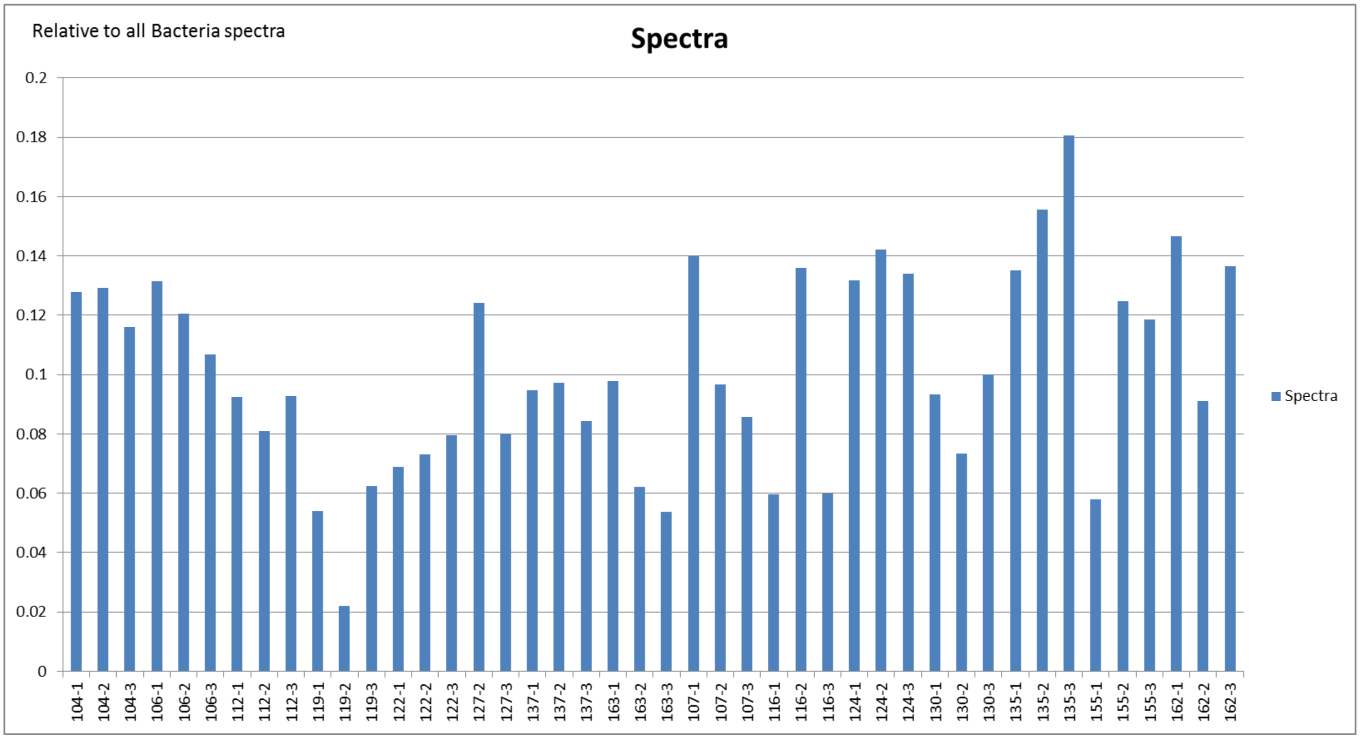

Supplement: S1 File — This file contains S4 Fig Hierarchical clustering of oligoprofiles, S5 Fig Distribution of butyrate and propionate synthesis, S6 Fig Clustering of Pearson correlations of bacterial/eukaryotic assignment ratios, S7 Fig A) Distribution of the 5 most abundant phyla for 16S rRNA gene and spectra per sample. B) Bacteroidetes qPCR results, S8 Fig Functionality (COG) across phyla, and S9 Fig Abundance and activity of F. prausnitzii. (PDF) [file pone.0153294.s004.pdf]
