# Supplementary material for: Faecal Metaproteomic Analysis Reveals a Personalized and Stable Functional Microbiome and Limited Effects of a Probiotic Intervention in Adults
Source: PLoS One. 2016 Apr 12;11(4):e0153294. doi: 10.1371/journal.pone.0153294 (PMC4829149; doi:10.1371/journal.pone.0153294)
Supplement: S2 Table — (PDF) [file pone.0153294.s006.pdf]

Table S2. Health status of study participants. The study participants were requested to fill out a health questionnaire with questions regarding their overall wellbeing. The self-reported health status is provided with health questionnaire information as being relevant for this study. Information on those subjects is provided and connected to our metaprotoemics data who reported regular probiotic usage, antibiotic treatment, and intestinal symptoms.

Health questionnaire	Subject identification number					
	104	116	119	155	162	163
Health status *	Good	Average	Good	Good	Good	Average
Regular probiotic usage	Y	Y	N	Y	N	N
Antibiotic before # sample	-	-	2	-	3	-
Intestinal symptom before # sample	2 (diarrhea)	-	2 (stomach ache)<br>3 (stomach ache, vomiting)	-	-	1 (diarrhea)<br>Constipation, vomiting

Association between health status and metaproteome

As we noted the high individuality of the metaproteome, we linked the metaproteomic data to the available information on the health status of the study participants (Table S2). The metaproteome data of subject 119 who had reported several intestinal symptoms during the course of the study and who had been treated with an antibiotic just before the sampling of TP 2 did exhibit some peculiarities. The samples of this subject were the most distant from the others when the peptide profiles of all the analysed samples were placed into hierarchical clusters i.e. the peptide profile of this metaproteome was the most individually distinctive one in the studied cohort (Fig 5). High levels of alkaline phosphatase were observed, xylose isomerase derived from Actinobacteria was missing, and there were similar amounts of the proteins involved in butyrate and propionate synthesis (also noted for subject 155).

When clustering the Jaccard similarities the samples 116-2 and 155-1 were relatively distant from the two other time points for these two individuals while for the other participants all three samples were more similar (except for 106-1). Interestingly, both subjects 116 and 155 stated that they were consuming

probiotics on a regular basis, and possibly have been affected by the restriction not to use dairy products that contained probiotics as part of the study protocol. In subject 104, who reported diarrhea during the intervention phase before the TP2 sample was collected, we found that the amount of butyryl-CoA dehydrogenase was reduced at TP2 in comparison to the two flanking samples. Participant 162 took the antibiotic penicillin V at the end of the study, one day before the third sample was taken; however, no obvious changes were observed in the metaproteome. In the samples of individual 163 who reported diarrhea throughout the whole placebo phase, then constipation, vomiting, and later again constipation the levels of alkaline phosphatase were higher than average, at 11%. In contrast, the levels of alpha-1-antitrypsin were rather low (3% versus 10%) and no serpin B was detected whereas this protein was present in all other individuals.

The samples of subject 135 who reported intestinal problems during the study (vomiting while taking the probiotic), were the second most distant when clustering the identified peptides and the most distant when clustering the oligonucleotide data. Almost 1% of peptides with phylum assignment were of Euryarchaea origin. Bacteria related to *Prevotella melaninogenica* and *Faecalibacterium prausnitzii* each represented about 20% of the microbiota before the intervention (TP1) and both declined below 5% during and after the intervention (time point two and three). Microarray signals for Bacteroidetes decreased from ~30% to less than 10%, with a smaller reduction at the peptide level. Concomitantly, bacteria related to Uncultured Clostridiales I increased from less than 1% (time point 1) to 11% (time point 2). These bacteria are related to *F. prausnitzii* and this may explain the large discrepancy between the microarray and peptide measurements found for *F. prausnitzii* (Fig 8). In general, it is possible that a different phylotype contributed to the measured protein activity than that specified due to microarray cross-hybridization (i.e. some phylotype other than target phylotype binding to the oligonucleotide probe) or to a bias in sequence database (i.e. species from which protein derived is missing in the database but a species with homologous proteins is present).

The decrease of Bacteroidetes signals of around 2-fold from time point one to the time point two where they remained at the lower level whereas peptide measurements only revealed a small decline might be indicative that the protein expression activity of Bacteroidetes might be independent of its presence. Similarly, in subject 116 who rated his/her health as only average, low levels (<10%) of Bacteroidetes signals were observed throughout the study but peptide measurements found very high levels (close to 40%) of Bacteroidetes peptides at TP2.
