# Supplementary material for: Faecal Metaproteomic Analysis Reveals a Personalized and Stable Functional Microbiome and Limited Effects of a Probiotic Intervention in Adults
Source: PLoS One. 2016 Apr 12;11(4):e0153294. doi: 10.1371/journal.pone.0153294 (PMC4829149; doi:10.1371/journal.pone.0153294)
Supplement: S3 Table — (DOCX) [file pone.0153294.s007.docx]

**Table S3: Composition of the in-house human intestinal metaproteome database HIMPdb***

| **No.** | **Description** | **Number of sequences** |
| --- | --- | --- |
| 1 | 594 bacterial genomes** | 1,850,744 |
| 2 | Translation of 124 metagenomes of European subjects  Qin et al., 2010, Nature, 464 (7285): 59-65 | 3,267,604 |
| 3 | 13 metagenomes of Japanese subjects  Kurokawa et al. , 2007, DNA Res 14, 169-181 | 600,752 |
| 4 | Human protein sequences from Integr8 and Genbank | 69,879 |
| 5 | Putative human protein sequences from Genbank | 116,718 |
| 6 | Protein sequences from plants | 247,371 |

*** As described in [14] with extension of database 1 and addition of database 5.**

****List of bacterial genomes (**source: <ftp://ftp.ncbi.nih.gov/genomes/Bacteria/and/Bacteria_DRAFT>; downloaded on 8/6/2011)

| **Species** | **Status** | **Proteins in db** |
| --- | --- | --- |
| Acidaminococcus_D21_uid55871 | draft | 2004 |
| Acidaminococcus_fermentans_DSM_20731_uid43471 | complete | 2021 |
| Acinetobacter_baumannii_AB900_uid55405 | draft | 3707 |
| Acinetobacter_junii_SH205_uid41585 | draft | 3183 |
| Acinetobacter_radioresistens_SH164_uid41589 | draft | 2866 |
| Actinobacillus_succinogenes_130Z_uid58247 | complete | 2079 |
| Akkermansia_muciniphila_ATCC_BAA_835_uid58985 | complete | 2136 |
| Alistipes_HGB5 | draft (manual) | 2947 |
| Alistipes_putredinis_DSM_17216_uid54803 | draft | 2737 |
| Alistipes_shahii_WAL_8301 | draft (manual) | 2553 |
| Anaerobaculum_hydrogeniformans_ATCC_BAA_1850_uid55759 | draft | 2492 |
| Anaerococcus_hydrogenalis_DSM_7454_uid55367 | draft | 2062 |
| Anaerococcus_prevotii_DSM_20548_uid59219 | complete | 1801 |
| Anaerofustis_stercorihominis_DSM_17244_uid54805 | draft | 2324 |
| Anaerostipes_3_2_56FAA_uid61867 | draft | 3366 |
| Anaerostipes_caccae_DSM_14662_uid54561 | draft | 3731 |
| Anaerotruncus_colihominis_DSM_17241_uid54807 | draft | 4396 |
| Arcobacter_butzleri_JV22_uid61483 | draft | 2376 |
| Bacillus_amyloliquefaciens_FZB42_uid58271 | complete | 3681 |
| Bacillus_anthracis__Ames_Ancestor__uid58083 | complete | 5420 |
| Bacillus_cereus_AH187_uid58753 | complete | 5732 |
| Bacillus_cereus_B4264_uid58757 | complete | 5362 |
| Bacillus_cereus_G9842_uid58759 | complete | 5800 |
| Bacillus_cytotoxicus_NVH_391_98_uid58317 | complete | 3826 |
| Bacillus_subtilis_168_uid57675 | complete | 4157 |
| Bacillus_weihenstephanensis_KBAB4_uid58315 | complete | 5605 |
| Bacteroides_1_1_14_uid49709 | draft | 4759 |
| Bacteroides_1_1_6_uid55577 | draft | 5572 |
| Bacteroides_20_3_uid50765 | draft | 4865 |
| Bacteroides_2_1_16_uid41707 | draft | 4592 |
| Bacteroides_2_1_22_uid41621 | draft | 4729 |
| Bacteroides_2_1_33B_uid41591 | draft | 3959 |
| Bacteroides_2_1_7_uid55579 | draft | 4358 |
| Bacteroides_2_2_4_uid55581 | draft | 5939 |
| Bacteroides_3_1_19_uid49705 | draft | 4243 |
| Bacteroides_3_1_23_uid49963 | draft | 4903 |
| Bacteroides_3_1_33FAA_uid41705 | draft | 4635 |
| Bacteroides_3_1_40A_uid62053 | draft | 4555 |
| Bacteroides_3_2_5_uid55583 | draft | 4492 |
| Bacteroides_4_1_36_uid61871 | draft | 3719 |
| Bacteroides_4_3_47FAA_uid55585 | draft | 4596 |
| Bacteroides_9_1_42FAA_uid55587 | draft | 4848 |
| Bacteroides_D1_uid55589 | draft | 4764 |
| Bacteroides_D20_uid42369 | draft | 3650 |
| Bacteroides_D22_uid49721 | draft | 4925 |
| Bacteroides_D2_uid55591 | draft | 5125 |
| Bacteroides_caccae_ATCC_43185_uid54521 | draft | 3830 |
| Bacteroides_capillosus_ATCC_29799_uid54531 | draft | 4745 |
| Bacteroides_cellulosilyticus_DSM_14838_uid55279 | draft | 5683 |
| Bacteroides_clarus_YIT_12056_uid66155 | draft | 3257 |
| Bacteroides_coprocola_DSM_17136_uid54879 | draft | 4271 |
| Bacteroides_coprophilus_DSM_18228_uid55301 | draft | 3818 |
| Bacteroides_coprosuis_DSM_18011_uid66921 | draft | 2386 |
| Bacteroides_dorei_5_1_36_D4_uid55593 | draft | 4421 |
| Bacteroides_dorei_DSM_17855_uid54993 | draft | 4923 |
| Bacteroides_eggerthii_1_2_48FAA_uid61869 | draft | 3855 |
| Bacteroides_eggerthii_DSM_20697_uid54989 | draft | 3696 |
| Bacteroides_finegoldii_DSM_17565_uid54985 | draft | 4463 |
| Bacteroides_fluxus_YIT_12057_uid66157 | draft | 3899 |
| Bacteroides_fragilis_3_1_12_uid55575 | draft | 4911 |
| Bacteroides_fragilis_NCTC_9343_uid57639 | complete | 4223 |
| Bacteroides_fragilis_YCH46_uid58195 | complete | 4616 |
| Bacteroides_helcogenes_P_36_108_uid62135 | complete | 3236 |
| Bacteroides_intestinalis_DSM_17393_uid54881 | draft | 4900 |
| Bacteroides_ovatus_ATCC_8483_uid54543 | draft | 5471 |
| Bacteroides_ovatus_SD_CC_2a_uid47359 | draft | 4904 |
| Bacteroides_ovatus_SD_CMC_3f_uid46973 | draft | 5268 |
| Bacteroides_pectinophilus_ATCC_43243_uid54987 | draft | 3219 |
| Bacteroides_plebeius_DSM_17135_uid54991 | draft | 3912 |
| Bacteroides_salanitronis_DSM_18170_uid63269 | complete | 3630 |
| Bacteroides_stercoris_ATCC_43183_uid54825 | draft | 3754 |
| Bacteroides_thetaiotaomicron_VPI_5482_uid62913 | complete | 4815 |
| Bacteroides_uniformis_ATCC_8492_uid54547 | draft | 4595 |
| Bacteroides_vulgatus_ATCC_8482_uid58253 | complete | 4062 |
| Bacteroides_vulgatus_PC510_uid47771 | draft | 3938 |
| Bacteroides_xylanisolvens_SD_CC_1b_uid47865 | draft | 4905 |
| Bacteroides_xylanisolvens_XB1A | draft (manual) | 4385 |
| Bacteroidetes_oral_taxon_274_F0058_uid49703 | draft | 1855 |
| Bartonella_quintana_Toulouse_uid57635 | complete | 1142 |
| Beijerinckia_indica_ATCC_9039_uid59057 | complete | 3779 |
| Bifidobacterium_12_1_47BFAA_uid61873 | draft | 2035 |
| Bifidobacterium_adolescentis_ATCC_15703_uid58559 | complete | 1632 |
| Bifidobacterium_adolescentis_L2_32_uid54549 | draft | 2398 |
| Bifidobacterium_angulatum_DSM_20098_uid55113 | draft | 1741 |
| Bifidobacterium_animalis_lactis_AD011_uid58911 | complete | 1521 |
| Bifidobacterium_animalis_lactis_BB12 | draft (manual) | 1628 |
| Bifidobacterium_animalis_lactis_Bl_04_uid59359 | complete | 1560 |
| Bifidobacterium_animalis_lactis_DSM_10140_uid59357 | complete | 1558 |
| Bifidobacterium_animalis_lactis_HN019_uid55085 | draft | 1563 |
| Bifidobacterium_animalis_lactis_V9 | draft (manual) | 1564 |
| Bifidobacterium_bifidum_NCIMB_41171_uid55283 | draft | 1840 |
| Bifidobacterium_bifidum_PRL2010_uid59883 | complete | 1704 |
| Bifidobacterium_bifidum_S17_uid59545 | complete | 1775 |
| Bifidobacterium_breve_DSM_20213_uid55111 | draft | 2236 |
| Bifidobacterium_catenulatum_DSM_16992_uid55369 | draft | 1930 |
| Bifidobacterium_dentium_ATCC_27678_uid54901 | draft | 2422 |
| Bifidobacterium_dentium_ATCC_27679_uid52349 | draft | 2328 |
| Bifidobacterium_dentium_Bd1_uid43091 | complete | 2117 |
| Bifidobacterium_dentium_JCVIHMP022_uid59471 | draft | 2267 |
| Bifidobacterium_gallicum_DSM_20093_uid55371 | draft | 1879 |
| Bifidobacterium_longum_BBMN68_uid60163 | complete | 1786 |
| Bifidobacterium_longum_DJO10A_uid58833 | complete | 1980 |
| Bifidobacterium_longum_JCM_1217_uid62695 | complete | 1912 |
| Bifidobacterium_longum_JDM301_uid49131 | complete | 1951 |
| Bifidobacterium_longum_NCC2705_uid57939 | complete | 1727 |
| Bifidobacterium_longum_infantis_157F_uid62693 | complete | 1986 |
| Bifidobacterium_longum_infantis_ATCC_15697_uid58677 | complete | 2410 |
| Bifidobacterium_longum_infantis_ATCC_55813_uid55465 | draft | 2093 |
| Bifidobacterium_longum_infantis_CCUG_52486_uid55285 | draft | 2227 |
| Bifidobacterium_longum_longum_F8 | draft (manual) | 1653 |
| Bifidobacterium_pseudocatenulatum_DSM_20438_uid55303 | draft | 2127 |
| Bilophila_wadsworthia_3_1_6_uid61875 | draft | 3645 |
| Blautia_hansenii_DSM_20583_uid55275 | draft | 3159 |
| Blautia_hydrogenotrophica_DSM_10507_uid54939 | draft | 3854 |
| Brachyspira_murdochii_DSM_12563_uid48819 | complete | 2803 |
| Brachyspira_pilosicoli_95_1000_uid50609 | complete | 2296 |
| Bryantella_formatexigens_DSM_14469_uid54943 | draft | 4855 |
| Burkholderia_oklahomensis_EO147_uid54791 | draft | 7044 |
| Burkholderiales_bacterium_1_1_47_uid51545 | draft | 2373 |
| Butyrivibrio_crossotus_DSM_2876_uid55091 | draft | 2521 |
| Butyrivibrio_fibrisolvens_16_4 | draft (manual) | 2860 |
| Butyrivibrio_proteoclasticus_B316_uid51489 | complete | 3804 |
| Caldicellulosiruptor_bescii_DSM_6725_uid59201 | complete | 2663 |
| Campylobacter_coli_JV20_uid51831 | draft | 1765 |
| Campylobacter_concisus_13826_uid58667 | complete | 1977 |
| Campylobacter_curvus_525_92_uid58669 | complete | 1928 |
| Campylobacter_hominis_ATCC_BAA_381_uid58981 | complete | 1681 |
| Campylobacter_jejuni_81_176_uid58503 | complete | 1748 |
| Campylobacter_jejuni_CG8486_uid54417 | draft | 1423 |
| Campylobacter_jejuni_HB93_13_uid54375 | draft | 1704 |
| Campylobacter_jejuni_NCTC_11168_uid57587 | complete | 1614 |
| Campylobacter_jejuni_doylei_269_97_uid58671 | complete | 1726 |
| Campylobacter_jejuni_jejuni_M1 | draft (manual) | 1618 |
| Campylobacter_upsaliensis_JV21_uid61485 | draft | 1704 |
| Campylobacter_upsaliensis_RM3195_uid54143 | draft | 1916 |
| Candidatus_Sulcia_muelleri_DMIN_uid47075 | complete | 226 |
| Catenibacterium_mitsuokai_DSM_15897_uid54829 | draft | 2967 |
| Citrobacter_30_2_uid55595 | draft | 4721 |
| Citrobacter_koseri_ATCC_BAA_895_uid58143 | complete | 4970 |
| Citrobacter_rodentium_ICC168_uid43089 | complete | 4890 |
| Citrobacter_youngae_ATCC_29220_uid55081 | draft | 5238 |
| Clostridiales_SM4_1 | draft (manual) | 1622 |
| Clostridiales_SS3_4 | draft (manual) | 2943 |
| Clostridiales_SSC_2 | draft (manual) | 2742 |
| Clostridiales_bacterium_1_7_47FAA_uid55287 | draft | 6217 |
| Clostridium_7_2_43FAA_uid55597 | draft | 3442 |
| Clostridium_D5_uid63427 | draft | 4487 |
| Clostridium_HGF2_uid61051 | draft | 4168 |
| Clostridium_L2_50_uid54559 | draft | 2917 |
| Clostridium_M62_1_uid54557 | draft | 4121 |
| Clostridium_SS2_1_uid54553 | draft | 3160 |
| Clostridium_acetobutylicum_ATCC_824_uid57677 | complete | 3844 |
| Clostridium_asparagiforme_DSM_15981_uid55115 | draft | 6767 |
| Clostridium_bartlettii_DSM_16795_uid54809 | draft | 2777 |
| Clostridium_beijerinckii_NCIMB_8052_uid58137 | complete | 5017 |
| Clostridium_bolteae_ATCC_BAA_613_uid54523 | draft | 7156 |
| Clostridium_botulinum_A2_Kyoto_uid59229 | complete | 3862 |
| Clostridium_cellulolyticum_H10_uid58709 | complete | 3383 |
| Clostridium_difficile_NAP07_uid49315 | draft | 3579 |
| Clostridium_difficile_NAP08_uid49121 | draft | 3753 |
| Clostridium_difficile_R20291_uid40921 | complete | 3503 |
| Clostridium_hathewayi_DSM_13479_uid55373 | draft | 7501 |
| Clostridium_hiranonis_DSM_13275_uid55075 | draft | 2265 |
| Clostridium_hylemonae_DSM_15053_uid55299 | draft | 3948 |
| Clostridium_kluyveri_DSM_555_uid58885 | complete | 3906 |
| Clostridium_leptum_DSM_753_uid54605 | draft | 3876 |
| Clostridium_methylpentosum_DSM_5476_uid55281 | draft | 3862 |
| Clostridium_nexile_DSM_1787_uid55077 | draft | 4112 |
| Clostridium_novyi_NT_uid58643 | complete | 2312 |
| Clostridium_perfringens_ATCC_13124_uid57901 | complete | 2868 |
| Clostridium_phytofermentans_ISDg_uid58519 | complete | 3894 |
| Clostridium_ramosum_DSM_1402_uid54811 | draft | 3153 |
| Clostridium_saccharolyticum_K10 | draft (manual) | 2985 |
| Clostridium_scindens_ATCC_35704_uid54533 | draft | 3970 |
| Clostridium_spiroforme_DSM_1552_uid54607 | draft | 2453 |
| Clostridium_sporogenes_ATCC_15579_uid54895 | draft | 3759 |
| Clostridium_symbiosum_WAL_14163_uid63097 | draft | 4678 |
| Clostridium_symbiosum_WAL_14673_uid63157 | draft | 4185 |
| Clostridium_tetani_E88_uid57683 | complete | 2436 |
| Clostridium_thermocellum_ATCC_27405_uid57917 | complete | 3187 |
| Collinsella_aerofaciens_ATCC_25986_uid54525 | draft | 2346 |
| Collinsella_intestinalis_DSM_13280_uid55125 | draft | 1779 |
| Collinsella_stercoris_DSM_13279_uid54813 | draft | 2512 |
| Coprobacillus_29_1_uid62161 | draft | 3840 |
| Coprobacillus_D7_uid55621 | draft | 3381 |
| Coprococcus_ART55_1 | draft (manual) | 2102 |
| Coprococcus_catus_GD_7 | draft (manual) | 2934 |
| Coprococcus_comes_ATCC_27758_uid54883 | draft | 3889 |
| Coprococcus_eutactus_ATCC_27759_uid54541 | draft | 2945 |
| Corynebacterium_ammoniagenes_DSM_20306_uid48813 | draft | 2623 |
| Desulfitobacterium_hafniense_Y51_uid58605 | complete | 5057 |
| Desulfovibrio_3_1_syn3_uid51597 | draft | 3243 |
| Desulfovibrio_desulfuricans_ATCC_27774_uid59213 | complete | 2349 |
| Desulfovibrio_piger_ATCC_29098_uid54519 | draft | 3092 |
| Desulfovibrio_vulgaris__Miyazaki_F__uid59089 | complete | 3174 |
| Dorea_formicigenerans_ATCC_27755_uid54513 | draft | 3254 |
| Dorea_longicatena_DSM_13814_uid54515 | draft | 2950 |
| Dysgonomonas_mossii_DSM_22836_uid67097 | draft | 3417 |
| Edwardsiella_tarda_ATCC_23685_uid47355 | draft | 3918 |
| Edwardsiella_tarda_FL6_60 | draft (manual) | 3244 |
| Eggerthella_1_3_56FAA_uid61877 | draft | 2930 |
| Eggerthella_HGA1_uid63585 | draft | 3012 |
| Elusimicrobium_minutum_Pei191_uid58949 | complete | 1529 |
| Enterobacter_cancerogenus_ATCC_35316_uid55079 | draft | 4603 |
| Enterobacter_cloacae_cloacae_NCTC_9394 | draft (manual) | 3685 |
| Enterobacteriaceae_bacterium_9_2_54FAA_uid61879 | draft | 4159 |
| Enterococcus_7L76 | draft (manual) | 2270 |
| Enterococcus_faecalis_PC1_1_uid46979 | draft | 2684 |
| Enterococcus_faecalis_TX0104_uid55351 | draft | 3261 |
| Enterococcus_faecalis_TX1302 | draft (manual) | 2847 |
| Enterococcus_faecalis_TX1322_uid55479 | draft | 2995 |
| Enterococcus_faecalis_TX1341 | draft (manual) | 3031 |
| Enterococcus_faecalis_TX1342 | draft (manual) | 2790 |
| Enterococcus_faecalis_TX1346 | draft (manual) | 2826 |
| Enterococcus_faecalis_TX1467 | draft (manual) | 3491 |
| Enterococcus_faecalis_TX2134_uid52587 | draft | 3188 |
| Enterococcus_faecalis_TX2137 | draft (manual) | 2992 |
| Enterococcus_faecalis_TX4244 | draft (manual) | 2897 |
| Enterococcus_faecalis_V583_uid57669 | complete | 3244 |
| Enterococcus_faecalis_X98_uid55711 | draft | 2801 |
| Enterococcus_faecium_1_141_733_uid55717 | draft | 2778 |
| Enterococcus_faecium_1_231_501_uid55715 | draft | 2747 |
| Enterococcus_faecium_Com12_uid55723 | draft | 2601 |
| Enterococcus_faecium_Com15_uid55725 | draft | 2721 |
| Enterococcus_faecium_DO_uid54089 | draft | 2713 |
| Enterococcus_faecium_E1071_uid47015 | draft | 2700 |
| Enterococcus_faecium_PC4_1 | draft (manual) | 2684 |
| Enterococcus_faecium_TX1330_uid55481 | draft | 2764 |
| Erysipelotrichaceae_bacterium_3_1_53_uid59459 | draft | 3857 |
| Erysipelotrichaceae_bacterium_5_2_54FAA_uid46995 | draft | 2874 |
| Escherichia_1_1_43_uid55599 | draft | 2238 |
| Escherichia_3_2_53FAA_uid55601 | draft | 5305 |
| Escherichia_4_1_40B_uid55603 | draft | 4606 |
| Escherichia_albertii_TW07627_uid55089 | draft | 4344 |
| Escherichia_coli_042 | draft (manual) | 4906 |
| Escherichia_coli_536_uid58531 | complete | 4601 |
| Escherichia_coli_APEC_O1_uid58623 | complete | 4851 |
| Escherichia_coli_ATCC_8739_uid58783 | complete | 4187 |
| Escherichia_coli_B7A_uid54297 | draft | 4596 |
| Escherichia_coli_E110019_uid54303 | draft | 4888 |
| Escherichia_coli_E24377A_uid58395 | complete | 4927 |
| Escherichia_coli_ED1a_uid59379 | complete | 4900 |
| Escherichia_coli_F11_uid54299 | draft | 4653 |
| Escherichia_coli_HS_uid58393 | complete | 4332 |
| Escherichia_coli_IHE3034 | draft (manual) | 4710 |
| Escherichia_coli_K_12_substr__DH10B_uid58979 | complete | 4106 |
| Escherichia_coli_K_12_substr__MG1655_uid57779 | complete | 4104 |
| Escherichia_coli_MS_107_1_uid50575 | draft | 5110 |
| Escherichia_coli_MS_110_3 | draft (manual) | 5386 |
| Escherichia_coli_MS_115_1_uid50627 | draft | 4995 |
| Escherichia_coli_MS_116_1_uid50635 | draft | 5126 |
| Escherichia_coli_MS_117_3 | draft (manual) | 5339 |
| Escherichia_coli_MS_119_7_uid50577 | draft | 5285 |
| Escherichia_coli_MS_124_1_uid50763 | draft | 5914 |
| Escherichia_coli_MS_145_7_uid59467 | draft | 5445 |
| Escherichia_coli_MS_146_1_uid50897 | draft | 4929 |
| Escherichia_coli_MS_153_1 | draft (manual) | 5419 |
| Escherichia_coli_MS_16_3 | draft (manual) | 5244 |
| Escherichia_coli_MS_175_1_uid50639 | draft | 4834 |
| Escherichia_coli_MS_182_1_uid50641 | draft | 5274 |
| Escherichia_coli_MS_185_1_uid50657 | draft | 5151 |
| Escherichia_coli_MS_187_1_uid50629 | draft | 4461 |
| Escherichia_coli_MS_196_1_uid50655 | draft | 5544 |
| Escherichia_coli_MS_198_1_uid50625 | draft | 5573 |
| Escherichia_coli_MS_200_1_uid50645 | draft | 5301 |
| Escherichia_coli_MS_21_1_uid50631 | draft | 5695 |
| Escherichia_coli_MS_45_1_uid50643 | draft | 5192 |
| Escherichia_coli_MS_57_2 | draft (manual) | 5190 |
| Escherichia_coli_MS_60_1 | draft (manual) | 5600 |
| Escherichia_coli_MS_69_1_uid50653 | draft | 5446 |
| Escherichia_coli_MS_78_1_uid50771 | draft | 4963 |
| Escherichia_coli_MS_84_1_uid50623 | draft | 5665 |
| Escherichia_coli_MS_85_1 | draft (manual) | 5945 |
| Escherichia_coli_O157_H7_EC508_uid54977 | draft | 4956 |
| Escherichia_coli_O157_H7_EDL933_uid57831 | complete | 5351 |
| Escherichia_coli_O157_H7_Sakai_uid57781 | complete | 5296 |
| Escherichia_coli_O26_H11_11368_uid41021 | complete | 5484 |
| Escherichia_coli_O55_H7_CB9615_uid46655 | complete | 5100 |
| Escherichia_coli_SE11_uid59425 | complete | 4976 |
| Escherichia_coli_SE15 | draft (manual) | 4477 |
| Escherichia_coli_UM146 | draft (manual) | 4746 |
| Escherichia_coli_UTI89_uid58541 | complete | 5136 |
| Eubacterium_biforme_DSM_3989_uid55117 | draft | 2512 |
| Eubacterium_cylindroides_T2_87 | draft (manual) | 1421 |
| Eubacterium_dolichum_DSM_3991_uid54609 | draft | 2335 |
| Eubacterium_eligens_ATCC_27750_uid59171 | complete | 2753 |
| Eubacterium_hallii_DSM_3353_uid54535 | draft | 3206 |
| Eubacterium_rectale_ATCC_33656_uid59169 | complete | 3593 |
| Eubacterium_rectale_DSM_17629 | draft (manual) | 2865 |
| Eubacterium_rectale_M104_1 | draft (manual) | 3174 |
| Eubacterium_siraeum_70_3 | draft (manual) | 2322 |
| Eubacterium_siraeum_DSM_15702_uid54603 | draft | 2683 |
| Eubacterium_siraeum_V10Sc8a | draft (manual) | 2184 |
| Eubacterium_ventriosum_ATCC_27560_uid54517 | draft | 2761 |
| Faecalibacterium_cf__prausnitzii_KLE1255_uid60645 | draft | 3331 |
| Faecalibacterium_prausnitzii_A2_165_uid54551 | draft | 3453 |
| Faecalibacterium_prausnitzii_L2_6 | draft (manual) | 2717 |
| Faecalibacterium_prausnitzii_M21_2_uid54555 | draft | 3450 |
| Faecalibacterium_prausnitzii_SL3_3 | draft (manual) | 2716 |
| Finegoldia_magna_ATCC_29328_uid58867 | complete | 1810 |
| Fusobacterium_1_1_41FAA_uid47783 | draft | 2325 |
| Fusobacterium_2_1_31_uid55605 | draft | 2295 |
| Fusobacterium_3_1_27_uid47791 | draft | 2006 |
| Fusobacterium_3_1_33_uid40851 | draft | 2120 |
| Fusobacterium_3_1_36A2_uid55995 | draft | 2129 |
| Fusobacterium_3_1_5R_uid55607 | draft | 1887 |
| Fusobacterium_4_1_13_uid55609 | draft | 2154 |
| Fusobacterium_7_1_uid55611 | draft | 2417 |
| Fusobacterium_D11_uid55627 | draft | 2327 |
| Fusobacterium_D12_uid55613 | draft | 2089 |
| Fusobacterium_gonidiaformans_ATCC_25563_uid55569 | draft | 1621 |
| Fusobacterium_mortiferum_ATCC_9817_uid55571 | draft | 2551 |
| Fusobacterium_nucleatum_ATCC_25586_uid57885 | complete | 2055 |
| Fusobacterium_ulcerans_ATCC_49185_uid55615 | draft | 3192 |
| Fusobacterium_varium_ATCC_27725_uid55573 | draft | 3012 |
| Gordonibacter_pamelaeae_7_10_1_bT | draft (manual) | 1998 |
| Grimontia_hollisae_CIP_101886_uid41501 | draft | 3540 |
| Helicobacter_bilis_ATCC_43879_uid55617 | draft | 2261 |
| Helicobacter_canadensis_MIT_98_5491_uid55359 | draft | 1532 |
| Helicobacter_cinaedi_CCUG_18818_uid55291 | draft | 2363 |
| Helicobacter_hepaticus_ATCC_51449_uid57737 | complete | 1864 |
| Helicobacter_pullorum_MIT_98_5489_uid55293 | draft | 1999 |
| Helicobacter_pylori_26695_uid57787 | complete | 1558 |
| Helicobacter_pylori_35A | draft (manual) | 1465 |
| Helicobacter_pylori_83 | draft (manual) | 1603 |
| Helicobacter_pylori_908 | draft (manual) | 1580 |
| Helicobacter_pylori_98_10_uid55307 | draft | 1522 |
| Helicobacter_pylori_B128_uid55309 | draft | 1722 |
| Helicobacter_pylori_Cuz20 | draft (manual) | 1556 |
| Helicobacter_pylori_G27_uid59305 | complete | 1502 |
| Helicobacter_pylori_HPAG1_uid58517 | complete | 1528 |
| Helicobacter_pylori_HPKX_438_AG0C1_uid55039 | draft | 2710 |
| Helicobacter_pylori_HPKX_438_CA4C1_uid55041 | draft | 3727 |
| Helicobacter_pylori_J99_uid57789 | complete | 1483 |
| Helicobacter_pylori_P12_uid59327 | complete | 1575 |
| Helicobacter_pylori_PeCan4_uid53539 | complete | 1555 |
| Helicobacter_pylori_SJM180_uid53541 | complete | 1572 |
| Helicobacter_pylori_Sat464 | draft (manual) | 1499 |
| Helicobacter_pylori_Shi470_uid59165 | complete | 1543 |
| Helicobacter_pylori_v225d | draft (manual) | 1537 |
| Helicobacter_winghamensis_ATCC_BAA_430_uid55619 | draft | 1636 |
| Holdemania_filiformis_DSM_12042_uid55297 | draft | 4192 |
| Klebsiella_1_1_55_uid46351 | draft | 5007 |
| Klebsiella_MS_92_3_uid66159 | draft | 6081 |
| Klebsiella_pneumoniae_342_uid59145 | complete | 5729 |
| Lachnospiraceae_bacterium_2_1_46FAA_uid66429 | draft | 2072 |
| Lachnospiraceae_bacterium_3_1_46FAA_uid66427 | draft | 2924 |
| Lachnospiraceae_bacterium_4_1_37FAA_uid63581 | draft | 2945 |
| Lachnospiraceae_bacterium_5_1_63FAA_uid61883 | draft | 3031 |
| Lachnospiraceae_bacterium_6_1_63FAA_uid66423 | draft | 2612 |
| Lachnospiraceae_bacterium_8_1_57FAA_uid61885 | draft | 2627 |
| Lachnospiraceae_bacterium_9_1_43BFAA_uid66425 | draft | 2707 |
| Lactobacillus_acidophilus_30SC_uid63605 | complete | 2053 |
| Lactobacillus_acidophilus_ATCC_4796_uid55489 | draft | 2014 |
| Lactobacillus_acidophilus_NCFM_uid57685 | complete | 1859 |
| Lactobacillus_amylolyticus_DSM_11664_uid48293 | draft | 1676 |
| Lactobacillus_amylovorus_GRL_1112_uid61179 | complete | 2113 |
| Lactobacillus_antri_DSM_16041_uid55491 | draft | 2213 |
| Lactobacillus_brevis_ATCC_367_uid57989 | complete | 2201 |
| Lactobacillus_brevis_gravesensis_ATCC_27305_uid55493 | draft | 3026 |
| Lactobacillus_buchneri_ATCC_11577_uid55495 | draft | 2987 |
| Lactobacillus_buchneri_NRRL_B_30929_uid66205 | complete | 2382 |
| Lactobacillus_casei_ATCC_334_uid57985 | complete | 2739 |
| Lactobacillus_casei_BL23_uid59237 | complete | 2969 |
| Lactobacillus_casei_Zhang_uid50673 | complete | 2828 |
| Lactobacillus_coleohominis_101_4_CHN_uid55977 | draft | 1649 |
| Lactobacillus_coryniformis_KCTC_3167_uid66991 | draft | 2724 |
| Lactobacillus_crispatus_125_2_CHN_uid55999 | draft | 2079 |
| Lactobacillus_crispatus_214_1_uid46981 | draft | 2150 |
| Lactobacillus_crispatus_CTV_05_uid60617 | draft | 2239 |
| Lactobacillus_crispatus_JV_V01_uid55355 | draft | 2203 |
| Lactobacillus_crispatus_MV_1A_US_uid55979 | draft | 2149 |
| Lactobacillus_crispatus_MV_3A_US_uid41363 | draft | 2328 |
| Lactobacillus_crispatus_ST1_uid48359 | complete | 2016 |
| Lactobacillus_delbrueckii_bulgaricus_ATCC_11842_uid58647 | complete | 1520 |
| Lactobacillus_delbrueckii_bulgaricus_ATCC_BAA_365_uid57987 | complete | 1682 |
| Lactobacillus_delbrueckii_bulgaricus_ND02_uid60621 | complete | 2004 |
| Lactobacillus_delbrueckii_bulgaricus_PB2003_044_T3_4_uid50571 | draft | 1896 |
| Lactobacillus_delbrueckii_lactis_DSM_20072 | draft (manual) | 1993 |
| Lactobacillus_fermentum_28_3_CHN_uid40899 | draft | 1875 |
| Lactobacillus_fermentum_ATCC_14931_uid55497 | draft | 1856 |
| Lactobacillus_fermentum_CECT_5716 | draft (manual) | 1050 |
| Lactobacillus_fermentum_IFO_3956_uid58865 | complete | 1838 |
| Lactobacillus_gasseri_202_4_uid55921 | draft | 1770 |
| Lactobacillus_gasseri_224_1_uid42955 | draft | 2241 |
| Lactobacillus_gasseri_ATCC_33323_uid57687 | complete | 1751 |
| Lactobacillus_gasseri_JV_V03_uid55499 | draft | 1976 |
| Lactobacillus_gasseri_MV_22_uid55431 | draft | 1617 |
| Lactobacillus_helveticus_DPC_4571_uid58761 | complete | 1610 |
| Lactobacillus_helveticus_DSM_20075_uid55883 | draft | 2064 |
| Lactobacillus_hilgardii_ATCC_8290_uid55501 | draft | 2775 |
| Lactobacillus_iners_AB_1_uid51205 | draft | 1207 |
| Lactobacillus_iners_ATCC_55195_uid61493 | draft | 1144 |
| Lactobacillus_iners_DSM_13335_uid55503 | draft | 1214 |
| Lactobacillus_iners_LEAF_2052A_d_uid60571 | draft | 1254 |
| Lactobacillus_iners_LEAF_2053A_b_uid60573 | draft | 1275 |
| Lactobacillus_iners_LEAF_2062A_h1_uid60569 | draft | 1260 |
| Lactobacillus_iners_LEAF_3008A_a_uid60567 | draft | 1206 |
| Lactobacillus_iners_LactinV_01V1_a_uid59479 | draft | 1521 |
| Lactobacillus_iners_LactinV_03V1_b_uid59477 | draft | 1457 |
| Lactobacillus_iners_LactinV_09V1_c_uid59475 | draft | 1356 |
| Lactobacillus_iners_LactinV_11V1_d_uid59473 | draft | 1335 |
| Lactobacillus_iners_SPIN_1401G_uid66145 | draft | 1235 |
| Lactobacillus_iners_SPIN_2503V10_D_uid59481 | draft | 1270 |
| Lactobacillus_iners_UPII_143_D_uid63609 | draft | 1182 |
| Lactobacillus_iners_UPII_60_B_uid63611 | draft | 1274 |
| Lactobacillus_jensenii_1153_uid55433 | draft | 1346 |
| Lactobacillus_jensenii_115_3_CHN_uid40897 | draft | 1469 |
| Lactobacillus_jensenii_208_1_uid43009 | draft | 3172 |
| Lactobacillus_jensenii_269_3_uid55867 | draft | 1570 |
| Lactobacillus_jensenii_27_2_CHN_uid55981 | draft | 1474 |
| Lactobacillus_jensenii_JV_V16_uid59525 | draft | 1448 |
| Lactobacillus_jensenii_SJ_7A_US_uid40901 | draft | 1627 |
| Lactobacillus_johnsonii_ATCC_33200_uid55505 | draft | 1833 |
| Lactobacillus_johnsonii_FI9785_uid41735 | complete | 1726 |
| Lactobacillus_johnsonii_NCC_533_uid58029 | complete | 1821 |
| Lactobacillus_oris_PB013_T2_3_uid60565 | draft | 2024 |
| Lactobacillus_paracasei_8700_2_uid55295 | draft | 3009 |
| Lactobacillus_paracasei_ATCC_25302_uid55519 | draft | 3023 |
| Lactobacillus_plantarum_ATCC_14917_uid55521 | draft | 3132 |
| Lactobacillus_plantarum_JDM1_uid59361 | complete | 2929 |
| Lactobacillus_plantarum_ST_III_uid53537 | complete | 3016 |
| Lactobacillus_plantarum_WCFS1_uid62911 | complete | 3047 |
| Lactobacillus_reuteri_100_23_uid54165 | draft | 2163 |
| Lactobacillus_reuteri_CF48_3A_uid55541 | draft | 2157 |
| Lactobacillus_reuteri_DSM_20016_uid58471 | complete | 1899 |
| Lactobacillus_reuteri_JCM_1112_uid58875 | complete | 1820 |
| Lactobacillus_reuteri_MM2_3_uid55885 | draft | 2037 |
| Lactobacillus_reuteri_MM4_1A_uid55517 | draft | 2090 |
| Lactobacillus_reuteri_SD2112_uid55357 | draft | 2258 |
| Lactobacillus_rhamnosus_GG_uid59313 | complete | 2904 |
| Lactobacillus_rhamnosus_HN001_uid55109 | draft | 2720 |
| Lactobacillus_rhamnosus_LMS2_1_uid55507 | draft | 3132 |
| Lactobacillus_rhamnosus_Lc_705_uid59315 | complete | 2944 |
| Lactobacillus_ruminis_ATCC_25644_uid55509 | draft | 2244 |
| Lactobacillus_sakei_23K_uid58281 | complete | 1857 |
| Lactobacillus_salivarius_ACS_116_V_Col5a_uid50761 | draft | 2115 |
| Lactobacillus_salivarius_ATCC_11741_uid55511 | draft | 1974 |
| Lactobacillus_salivarius_CECT_5713 | draft (manual) | 1552 |
| Lactobacillus_salivarius_UCC118_uid58233 | complete | 2006 |
| Lactobacillus_ultunensis_DSM_16047_uid55513 | draft | 2203 |
| Lactobacillus_vaginalis_ATCC_49540_uid55515 | draft | 1866 |
| Lactococcus_lactis_Il1403_uid57671 | complete | 2312 |
| Lactococcus_lactis_KF147_uid42831 | complete | 2468 |
| Lactococcus_lactis_cremoris_MG1363_uid58837 | complete | 2426 |
| Lactococcus_lactis_cremoris_SK11_uid57983 | complete | 2489 |
| Laribacter_hongkongensis_HLHK9_uid59265 | complete | 3207 |
| Lawsonia_intracellularis_PHE_MN1_00_uid61575 | complete | 1340 |
| Legionella_pneumophila_Corby_uid58733 | complete | 3184 |
| Leptospira_interrogans_serovar_Lai_56601_uid57881 | complete | 3697 |
| Leuconostoc_mesenteroides_cremoris_ATCC_19254_uid55887 | draft | 1832 |
| Listeria_grayi_DSM_20601_uid55523 | draft | 2622 |
| Listeria_innocua_Clip11262_uid61567 | complete | 3040 |
| Listeria_monocytogenes_08_5923_uid43727 | complete | 2956 |
| Listeria_monocytogenes_10403S_uid54461 | draft | 2845 |
| Listeria_monocytogenes_EGD_e_uid61583 | complete | 2843 |
| Listeria_monocytogenes_F6900_uid54445 | draft | 3001 |
| Listeria_monocytogenes_FSL_F2_515_uid54421 | draft | 2622 |
| Listeria_monocytogenes_FSL_J1_175_uid54425 | draft | 3120 |
| Listeria_monocytogenes_FSL_J1_194_uid54423 | draft | 3009 |
| Listeria_monocytogenes_FSL_J1_208_uid54427 | draft | 2718 |
| Listeria_monocytogenes_FSL_J2_003_uid54429 | draft | 2901 |
| Listeria_monocytogenes_FSL_J2_064_uid54433 | draft | 2900 |
| Listeria_monocytogenes_FSL_J2_071_uid54431 | draft | 2713 |
| Listeria_monocytogenes_FSL_N1_017_uid54435 | draft | 3166 |
| Listeria_monocytogenes_FSL_N3_165_uid54437 | draft | 2888 |
| Listeria_monocytogenes_FSL_R2_503_uid54439 | draft | 3021 |
| Listeria_monocytogenes_FSL_R2_561_uid54441 | draft | 2927 |
| Listeria_monocytogenes_Finland_1988_uid54443 | draft | 2704 |
| Listeria_monocytogenes_HCC23_uid59203 | complete | 2969 |
| Listeria_monocytogenes_HPB2262_uid54465 | draft | 3046 |
| Listeria_monocytogenes_J0161_uid54459 | draft | 3070 |
| Listeria_monocytogenes_J2818_uid54447 | draft | 3071 |
| Listeria_monocytogenes_LO28_uid54449 | draft | 2954 |
| Listeria_monocytogenes_serotype_1_2a_F6854_uid54127 | draft | 2955 |
| Listeria_monocytogenes_serotype_4b_F2365_uid57689 | complete | 2812 |
| Listeria_monocytogenes_serotype_4b_H7858_uid54125 | draft | 3099 |
| Mannheimia_succiniciproducens_MBEL55E_uid58197 | complete | 2348 |
| Megamonas_hypermegale_ART12_1 | draft (manual) | 2079 |
| Methanobrevibacter_ruminantium_M1_uid45857 | complete | 2199 |
| Methanobrevibacter_smithii_ATCC_35061_uid58827 | complete | 1782 |
| Methanobrevibacter_smithii_DSM_2374_uid55123 | draft | 1710 |
| Methanobrevibacter_smithii_DSM_2375_uid54983 | draft | 1701 |
| Methanosphaera_stadtmanae_DSM_3091_uid58407 | complete | 1530 |
| Methanothermobacter_thermautotrophicus_Delta_H_uid57877 | complete | 1865 |
| Mitsuokella_multacida_DSM_20544_uid55073 | draft | 2544 |
| Moorella_thermoacetica_ATCC_39073_uid58051 | complete | 2461 |
| Mycobacterium_avium_paratuberculosis_K_10_uid57699 | complete | 4345 |
| Mycobacterium_bovis_BCG_Pasteur_1173P2_uid58781 | complete | 3932 |
| Mycoplasma_hominis_uid41875 | complete | 523 |
| Neisseria_gonorrhoeae_NCCP11945_uid59191 | complete | 2647 |
| Neisseria_meningitidis_053442_uid58587 | complete | 2012 |
| Opitutaceae_bacterium_TAV2_uid54579 | draft | 4787 |
| Oxalobacter_formigenes_HOxBLS_uid55623 | draft | 2122 |
| Oxalobacter_formigenes_OXCC13_uid55625 | draft | 2075 |
| Paenibacillus_HGF5_uid66147 | draft | 6462 |
| Paenibacillus_HGF7 | draft (manual) | 5953 |
| Paenibacillus_JDR_2_uid59021 | complete | 6189 |
| Parabacteroides_D13_uid55997 | draft | 4488 |
| Parabacteroides_distasonis_ATCC_8503_uid58301 | complete | 3849 |
| Parabacteroides_johnsonii_DSM_18315_uid55269 | draft | 4487 |
| Parabacteroides_merdae_ATCC_43184_uid54545 | draft | 4322 |
| Paraprevotella_xylaniphila_YIT_11841_uid66381 | draft | 3423 |
| Parasutterella_excrementihominis_YIT_11859 | draft (manual) | 2739 |
| Parvimonas_micra_ATCC_33270_uid54527 | draft | 1671 |
| Pediococcus_acidilactici_7_4_uid42365 | draft | 1838 |
| Pediococcus_acidilactici_DSM_20284_uid51711 | draft | 1880 |
| Phascolarctobacterium_YIT_12067_uid62745 | draft | 2145 |
| Porphyromonas_gingivalis_ATCC_33277_uid58879 | complete | 2075 |
| Porphyromonas_gingivalis_W83_uid57641 | complete | 1902 |
| Prevotella_copri_DSM_18205_uid55277 | draft | 3314 |
| Prevotella_ruminicola_23_uid47507 | complete | 2752 |
| Prevotella_salivae_DSM_15606_uid61887 | draft | 2908 |
| Propionibacterium_acnes_KPA171202_uid58101 | complete | 2288 |
| Proteus_mirabilis_HI4320_uid61599 | complete | 3657 |
| Proteus_penneri_ATCC_35198_uid54897 | draft | 4864 |
| Providencia_alcalifaciens_DSM_30120_uid55119 | draft | 4002 |
| Providencia_rettgeri_DSM_1131_uid55121 | draft | 4718 |
| Providencia_rustigianii_DSM_4541_uid55071 | draft | 3947 |
| Providencia_stuartii_ATCC_25827_uid54899 | draft | 4697 |
| Pseudomonas_aeruginosa_LESB58_uid59275 | complete | 5919 |
| Pseudomonas_fluorescens_Pf_5_uid57937 | complete | 6128 |
| Pseudomonas_putida_KT2440_uid57843 | complete | 5328 |
| Ralstonia_5_7_47FAA_uid59461 | draft | 4776 |
| Rickettsia_rickettsii__Sheila_Smith__uid58027 | complete | 1321 |
| Roseburia_intestinalis_L1_82_uid55267 | draft | 4671 |
| Roseburia_intestinalis_M50_1 | draft (manual) | 3418 |
| Roseburia_intestinalis_XB6B4 | draft (manual) | 3551 |
| Roseburia_inulinivorans_DSM_16841_uid55375 | draft | 4390 |
| Roseobacter_denitrificans_OCh_114_uid58597 | complete | 4094 |
| Ruminococcaceae_bacterium_D16_uid52825 | draft | 3167 |
| Ruminococcus_18P13 | draft (manual) | 2092 |
| Ruminococcus_5_1_39BFAA_uid55629 | draft | 3511 |
| Ruminococcus_SR1_5 | draft (manual) | 3192 |
| Ruminococcus_albus_7_uid51721 | complete | 3860 |
| Ruminococcus_albus_8_uid47357 | draft | 3822 |
| Ruminococcus_bromii_L2_63 | draft (manual) | 1800 |
| Ruminococcus_flavefaciens_FD_1_uid55965 | draft | 4134 |
| Ruminococcus_gnavus_ATCC_29149_uid54537 | draft | 3876 |
| Ruminococcus_lactaris_ATCC_29176_uid54903 | draft | 2729 |
| Ruminococcus_obeum_A2_162 | draft (manual) | 3115 |
| Ruminococcus_obeum_ATCC_29174_uid54509 | draft | 4119 |
| Ruminococcus_torques_ATCC_27756_uid54511 | draft | 2845 |
| Ruminococcus_torques_L2_14 | draft (manual) | 2763 |
| Salmonella_enterica_serovar_Typhimurium_LT2_uid57799 | complete | 4508 |
| Sebaldella_termitidis_ATCC_33386_uid41865 | complete | 4144 |
| Serratia_proteamaculans_568_uid58725 | complete | 4933 |
| Shigella_D9_uid55631 | draft | 4680 |
| Slackia_heliotrinireducens_DSM_20476_uid59051 | complete | 2751 |
| Staphylococcus_aureus_JH9_uid58455 | complete | 2719 |
| Staphylococcus_carnosus_TM300_uid59401 | complete | 2453 |
| Streptobacillus_moniliformis_DSM_12112_uid41863 | complete | 1441 |
| Streptococcus_2_1_36FAA_uid41507 | draft | 2127 |
| Streptococcus_agalactiae_2603V_R_uid57943 | complete | 2114 |
| Streptococcus_anginosus_1_2_62CV_uid62163 | draft | 1763 |
| Streptococcus_equinus_ATCC_9812_uid62297 | draft | 1787 |
| Streptococcus_infantarius_ATCC_BAA_102_uid54885 | draft | 2045 |
| Streptococcus_mitis_B6_uid46097 | complete | 2000 |
| Streptococcus_mutans_UA159_uid57947 | complete | 1937 |
| Streptococcus_pneumoniae_Hungary19A_6_uid59117 | complete | 2142 |
| Streptococcus_pyogenes_MGAS10750_uid58575 | complete | 1970 |
| Streptococcus_thermophilus_LMD_9_uid58327 | complete | 1685 |
| Streptococcus_uberis_0140J_uid57959 | complete | 1759 |
| Subdoligranulum_variabile_DSM_15176_uid54539 | draft | 3360 |
| Succinatimonas_hippei_YIT_12066_uid62747 | draft | 2164 |
| Sutterella_wadsworthensis_3_1_45B_uid62165 | draft | 2370 |
| Symbiobacterium_thermophilum_IAM_14863_uid58165 | complete | 3326 |
| Syntrophobacter_fumaroxidans_MPOB_uid58177 | complete | 4063 |
| Turicibacter_HGF1_uid63587 | draft | 2797 |
| Turicibacter_PC909_uid46977 | draft | 2767 |
| Variovorax_paradoxus_S110_uid59437 | complete | 6262 |
| Veillonella_3_1_44_uid47845 | draft | 1812 |
| Veillonella_6_1_27_uid47835 | draft | 1837 |
| Veillonella_parvula_DSM_2008_uid41927 | complete | 1839 |
| Vibrio_RC341_uid41105 | draft | 3504 |
| Vibrio_cholerae_CT_5369_93_uid41421 | draft | 2850 |
| Vibrio_cholerae_INDRE_91_1_uid41407 | draft | 3332 |
| Vibrio_cholerae_MZO_2_uid54587 | draft | 3376 |
| Vibrio_cholerae_O1_biovar_El_Tor_N16961_uid57623 | complete | 3781 |
| Vibrio_cholerae_RC27_uid41411 | draft | 3420 |
| Vibrio_cholerae_V51_uid54329 | draft | 2466 |
| Vibrio_cholerae_V52_uid54331 | draft | 3789 |
| Vibrio_furnissii_CIP_102972_uid41027 | draft | 4393 |
| Vibrio_mimicus_MB_451_uid41415 | draft | 3754 |
| Victivallis_vadensis_ATCC_BAA_548_uid54305 | draft | 4052 |
| Weissella_paramesenteroides_ATCC_33313_uid55901 | draft | 1949 |
| Wolinella_succinogenes_DSM_1740_uid61591 | complete | 2041 |
| Xanthobacter_autotrophicus_Py2_uid58453 | complete | 5028 |
| Xanthomonas_campestris_vesicatoria_85_10_uid58321 | complete | 4719 |
| Xenorhabdus_bovienii_SS_2004_uid46345 | complete | 4204 |
| Yersinia_bercovieri_ATCC_43970_uid54343 | draft | 3831 |
| Yersinia_pseudotuberculosis_IP_31758_uid58487 | complete | 4272 |
| Yersinia_pseudotuberculosis_IP_32953_uid58157 | complete | 4023 |
| Yersinia_rohdei_ATCC_43380_uid55247 | draft | 3798 |
